# Deprescribing antihypertensive medications in older people: a systematic review and a meta-analysis

**DOI:** 10.1186/s12877-025-06941-2

**Published:** 2026-01-05

**Authors:** Carmen Floriani, Giovanni Minchio, Angela Edith Schulthess-Lisibach, Carina Lundby, Maja Josephine Lundberg Andersen, Martina Zangger, Orestis Efthimiou, Enriqueta Vallejo-Yagüe, Stefan Neuner-Jehle, Wade Thompson, Jens Søndergaard, Lisa M. McCarthy, Carole Lunny, Rosalinde K. E. Poortvliet, Jacobijn Gussekloo, Stella S. Daskalopoulou, Marc von Gernler, Sven Streit

**Affiliations:** 1 Institute of Primary Health Care (BIHAM), University of Bern, Mittelstrasse 43, Bern, 3012 Switzerland; 2 University of Trento, Trento, Italy; 3 Institute of Social and Preventive Medicine (ISPM), University of Bern, Bern, Switzerland; 4 Hospital Pharmacy Funen, Odense University Hospital, Odense C, Denmark; 5 Department of Public Health, Clinical Pharmacology, Pharmacy and Environmental Medicine, University of Southern Denmark, Odense M, Denmark; 6 Institute of Primary Care, University Hospital Zurich, University of Zurich, Zurich, Switzerland; 7 Center for Primary and Community Care, University of Luzern, Luzern, Switzerland; 8 Department of Anesthesiology, Pharmacology and Therapeutics, Faculty of Medicine, University of British Columbia, Vancouver, Canada; 9 Department of Public Health, Research Unit of General Practice, University of Southern Denmark, Odense C, Denmark; 10 Department of Family and Community Medicine, Leslie Dan Faculty of Pharmacy, University of Toronto, Toronto, Canada; 11 Institute for Better Health, Trillium Health Partners, Mississauga, Canada; 12 Department of Anesthesiology, Pharmacology and Therapeutics, The University of British Columbia, Vancouver, Canada; 13 Department of Public Health and Primary Care, Leiden University Medical Center, Leiden, the Netherlands; 14 Department of Internal Medicine, Leiden University Medical Center, Leiden, the Netherlands; 15 LUMC Center for Medicine for Older People, Leiden University Medical Center, Leiden, The Netherlands; 16 Department of Medicine, Research Institute of the McGill University Health Centre, McGill University, Montreal, QC Canada; 17 Medical Library, University Library of Bern, University of Bern, Bern, Switzerland

**Keywords:** Deprescribing, Antihypertensive treatment, Older adults

## Abstract

**Introduction:**

Hypertension is highly prevalent among older people, and the balance of benefit and harm of antihypertensive therapy may shift with age. In certain cases, reducing or discontinuing antihypertensive treatment (deprescribing) may be appropriate. This systematic review and meta-analysis aimed to summarize available evidence on deprescribing antihypertensive medications in older adults aged 65 years and older.

**Methods:**

We searched MEDLINE, Embase, CINAHL, the Cochrane Library, the Web of Science Core Collection, ClinicalTrials.gov, ICTRP and Epistemonikos from inception to July 2024. We included randomized controlled trials (RCTs) and comparative observational studies (OS) comparing deprescribing versus continuation of antihypertensive medications in adults ≥ 65 years. The primary outcome was all-cause mortality. Secondary outcomes included myocardial infarction, heart failure, stroke, major adverse cardiovascular events (MACE), orthostatic hypotension and falls. Where possible, data were synthesized using meta-analysis to estimate odds ratios (ORs) and 95% Confidence Intervals (CI). We assessed the risk of bias in the RCTs in Covidence basing on the Cochrane Risk Of Bias (Rob 2) tool. For the observational studies we used the Newcastle Ottawa Scale for comparative observational studies.

**Results:**

We included 17 studies. Results from the observational studies are only reported as narrative summary. The pooled OR for all-cause mortality was 1.11 (95% CI 0.82–1.50; 6 RCTs). For secondary outcomes, pooled ORs were 1.32 (95% CI 0.30–5.92) for myocardial infarction (3 RCTs), 3.16 (95% CI 1.53–6.55) for heart failure (3 RCTs), and 3.08 (95% CI 0.73-13.00) for stroke (4 RCTs).

**Conclusion:**

The effects of deprescribing antihypertensive medications in older adults remain uncertain. The limited and low-event-rate evidence on key cardiovascular outcomes for older individuals highlights the need for individualized decision-making, especially in frail and multimorbid populations. This review provides a foundation for future research to address gaps and guide safer deprescribing practices in older adults in routine clinical practice.

**Supplementary Information:**

The online version contains supplementary material available at 10.1186/s12877-025-06941-2.

## Introduction

Hypertension is one of the main risk factors for cardiovascular events, such as stroke, myocardial infarction, and heart failure [1]. It increases from 27% in people younger than 60 years to 74% in people aged over 80 years [2].

The benefits of antihypertensive drugs are well-established, particularly in people at high risk for cardiovascular disease [3]. However, potential side effects may negatively impact overall health, especially in people over 75 years of age who often present with multiple comorbidities and polypharmacy [4]. Large randomized controlled trials (RCTs) have demonstrated that antihypertensive medications reduce the risk of cardiovascular events and mortality in older adults, including those aged 80 and above [1]. However, such trials often exclude older patients with multimorbidity and frailty, limiting the generalizability of their findings to this vulnerable population [5]. In certain circumstances, it may be appropriate to reduce or discontinue some medications to avoid adverse effects or minimize the overall treatment burden [6]. Regularly reassessing the benefits and harms or risks of antihypertensive drugs and considering the evidence supporting dose reduction or withdrawal can characterize good clinical practice [7]. Deprescribing is defined as the supervised withdrawal or dose reduction of medications when they are deemed no longer appropriate [8]. In routine clinical practice, deprescribing remains underutilized due to barriers related to patients, healthcare providers, and the broader healthcare system [9].

With this systematic review and meta-analysis, we aim to summarize the existing evidence on deprescribing anti-hypertensive drugs in older adults. Other published systematic reviews did not find evidence that discontinuing antihypertensive medications for primary prevention of cardiovascular disease had an effect on all-cause mortality and myocardial infarction in older adults [10]. A comprehensive update 2025 of the same review could confirm this conclusion with uncertain evidence about the effect of discontinuing antihypertensives [11]. Another review on deprescribing diuretic therapy found that it could be a safe and feasible option for carefully selected patients depending on the clinical setting [12]. Unlike these two reviews, our systematic review specifically targets adults aged 65 years and older, which aligns with standards in ageing and hypertension research. In addition, our review includes individuals both with and without a prior history of cardiovascular disease, thereby providing broader perspective on treatment effects in older populations. We also consider all classes of antihypertensives and draw on real-world evidence by using pragmatic outcomes from both randomized studies and observational research. This approach allows for a more comprehensive understanding of antihypertensive therapy in routine clinical practice among older adults.

## Methods

We conducted the systematic review in accordance with the Cochrane Handbook of Systematic Reviews of Interventions [13]. The review is reported according to the Preferred Reporting Items for Systematic Reviews and Meta-analyses (PRISMA) [14]. The PRISMA checklist is available under supplementary materials. This study was registered with PROSPERO (CRD42022313626, June 2022), deviations to the registered protocol are available in Appendix Sect. 1.

### Eligibility criteria

We included RCTs and comparative observational studies (OS) involving people 65 years and over, treated with antihypertensive medications. We placed no limits on study type, publication year or any other formal criteria. We considered only publications in English.

Studies conducted in various healthcare settings (outpatient, inpatient, and long-term care) were included. Antihypertensive medications of interest comprised diuretics (loop diuretics, thiazide-type diuretics, potassium-sparing diuretics, aldosterone receptor antagonists), beta-blockers, angiotensin converting enzyme inhibitors (ACE inhibitors), calcium channel blockers (CCBs), angiotensin II receptor blockers (ARB), and renin-angiotensin-aldosterone system inhibitors (RAASi).

Deprescribing of antihypertensive drugs was defined as the abrupt discontinuation, withdrawal, or dose reduction of an antihypertensive drug.

### Literature search

To identify all potentially relevant studies, an information specialist (M.vG.) searched MEDLINE (Ovid), Embase (Ovid), CINAHL, the Cochrane Library, the Web of Science Core Collection (Clarivate), ClinicalTrials.gov (NLM), ICTRP (WHO), and Epistemonikos (Epistemonikos Foundation) from inception to July 2024.

The search strategies for MEDLINE and Embase are based on Reeve et al. [10] and were further developed by the information specialist (M.vG.). The strategies were tested against a list of core references. After refinement, search strategies were set up for each information source based on database-specific controlled vocabulary and text words. Synonyms, acronyms, drug names and similar terms were included in the text word search. The detailed final search strategies are presented in Appendix Sect. 2. In addition to electronic database searching, reference lists and bibliographies from published RCTs and OS were searched for eligible studies.

### Study outcomes

The primary outcome was all-cause mortality. Secondary outcomes were myocardial infarction, heart failure, stroke, Major Adverse Cardiovascular Events (MACE), orthostatic hypotension, falls. An outcome summary table and a glossary of all subjective endpoints are in Appendix Sects. 3 and 4. All outcomes were considered irrespective of the duration of follow-up.

### Study selection

All identified citations were imported into the systematic review tool Covidence (Covidence systematic review software, Veritas Health Innovation, Melbourne, Australia) for screening and duplicates were removed. Titles and abstracts were independently screened by authors C.F., C.L. and M.Z. Any disagreements were resolved through consensus or consultation with a fourth reviewer (W.T.).

Full texts of potentially eligible studies were then independently assessed by authors C.F., A.E.SL. and M.A. Disagreements were resolved through consensus or by consulting a fourth reviewer (C.L.).

### Data collection and management

Data extraction was piloted on three studies by C.F. and C.L. and refined before full extraction to ensure consistency between the reviewers. The process was performed independently by authors C.F. and A.E.SL., any discrepancies were discussed and resolved by consensus. Extracted data included study characteristics (author, country, journal, funding, setting, design and participants number), patient characteristics (age and sex of participants, details of antihypertensive treatment), study intervention/exposure and comparator. Further, information on primary and secondary outcomes with main estimate, and duration of follow-up were collected. All results compatible with each outcome domain were extracted.

### Data synthesis and measure of intervention effect

When studies had comparable interventions (or exposures), outcomes, and populations, we performed a meta-analysis to estimate the overall effect size and direction, and to explore heterogeneity. Given that the dichotomous outcomes of interest are relatively rare (with incidence rates ranging from 0.5% to 10%), we used synthesized the Mantel Haenszel method as our primary analytical approach to calculate odds ratios (ORs) and the corresponding 95% Confidence Interval (CI) [15–17].

We presented results in forest plots. For visualization purposes, in studies with zero events in one of the two arms we used a non-fixed continuity correction [15].

We assessed studies’ statistical heterogeneity using the $\:{I}^{2}$ statistic and prediction intervals [18], based on an inverse-variance random effects model.

Only data from RCTs were included in the pooled quantitative synthesis, namely the meta-analysis. Results from the observational studies are only reported as narrative summary.

To address possible heterogeneity, we performed subgroup analysis based on the duration of the follow-up (≤ 6 months vs. >6 months) (Appendix Sect. 5).

As sensitivity analysis we performed random-effects, meta-analysis using Empirical Bayes method for all outcomes (Appendix Sect. 6) [19].

All analyses were carried out using STATA (version 18.0), using the commands in the ‘meta’ environment (StataCorp. 2023. Stata Statistical Software: Release 18. College Station, TX: StataCorp LLC).

### Assessment of risk of bias

The risk of bias in included studies was assessed independently by authors C.F. and A.E.SL. We assessed the risk of bias in the RCTs in Covidence basing on the Cochrane Risk Of Bias (Rob 2) tool [20]. For the observational studies we used the Newcastle Ottawa Scale for comparative observational studies [21]. Any disagreements were resolved through consensus or by consulting a third reviewer (C.L.).

### Assessment of small study effects and publication bias

We assessed the possibility of publication bias or small study effects by using a funnel plot and by doing a Harbord’s test [22] for binary outcomes (Appendix Sect. 7).

## Results

### Studies selection and characteristics

**Fig. 1:**
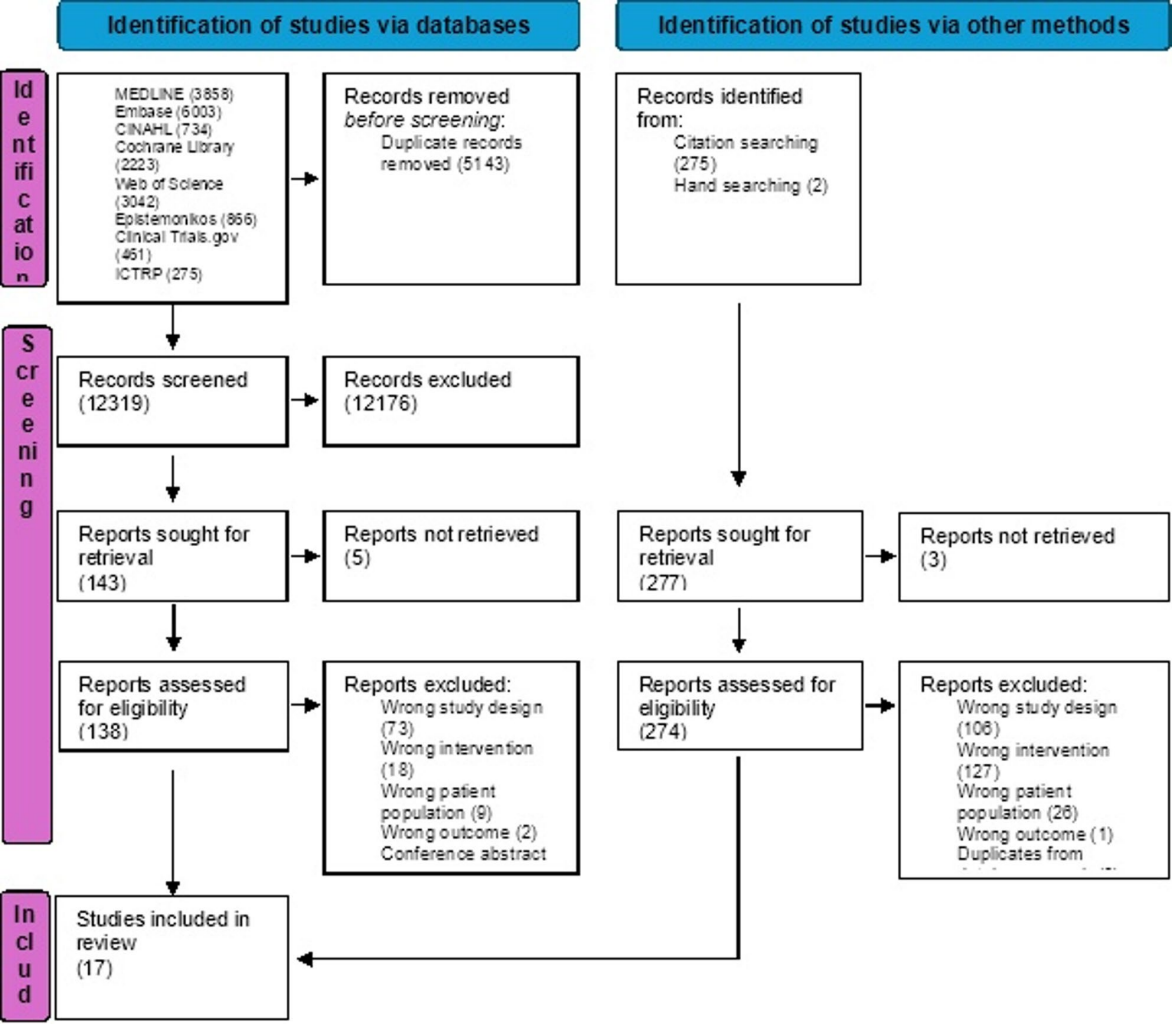
Study flow chart. Study flow chart showing all phases of the process from identification to inclusion

We identified 17,462 studies through the electronic search. After removing duplicates, C.F. C.L., and M.Z. screened 12,319 titles and abstracts. Of 138 full-text studies reviewed, 15 were included. Additionally, 277 articles were assessed for eligibility after reference checking, 2 of them fulfilled the eligibility criteria and were included **(**Fig. 1**)**.

Characteristics of the studies included are reported in Tables 1 and 2.. All RCTs were parallel group RCTs, and all the observational studies were cohort studies. Study duration and follow up period varied between twelve weeks [23] and 4 years for RCTs [24]. For observational studies follow up ranged from thirty days [25] to 8 years [26]. The sample size ranged from 77 patients [27] to 569 for RCTs [24], and from 1,541 [26] to 10,254 patients [28] for observational studies. The mean age of the study participants ranged from 68 years [29] to 85 years [30]. Most studies were conducted in a primary care setting ( [26, 30–32]), while one study included multiple settings, such as pharmacies, primary care setting and hospitals [25]. Two studies were carried out in long-term geriatric institutions/nursing homes [27, 33], while two others were conducted in hospital settings [23, 34], including one specifically in a geriatric ward [23].

**Table 1:** Chracteristics of randomized controlled trials

AuthorYear	Country	Setting	Study Design	N	Age(years)	Sex	Systolic BP (mmHg)(mean, at baseline)	Diastolic BP (mmHg)(mean, at baseline)	AntihypertensiveTreatment	InclusionCriteria	Exclusion criteria	Outcomes	FUP	
Burr 1977 [23]	UK	Ger ward	RCT	Overall106 Disc54Cont52	Disc81.6Cont82.4	Female93Male13	Disc 126.4Cont 128.6	Disc75.2 Cont78.2	Furosemide,Frusemide and ModureticModureticNavidrex KClopamideChlorthalidone	Diuretic prescription for more than 1 month	Congestive cardiac failure, ever had left ventricular failure, hypertension controlled in hospital by diuretic therapy, receiving diuretics for nephrotic syndrome/glaucoma	Number of Deaths, congestive heart failure, Blood pressure change, ankle oedema change, plasma potassium level, plasma urea level	12 W	
Myers 1982 [27]	CA	Ger instit	RCT	Overall77 Disc38 Cont39	Overall80.0	Female17Male60	Disc132 Cont130	Disc74 Cont68	Hydrochlorothiazid, Furosemide, Spironolactone, Hydrochlorothiazide and spironolactone Hydrochlorothiazide and triamterene	Diuretic use	Concurrent digoxin clinical or radiological evidence of heart failureresidents with hypertension	Number of Deaths,congestive heart failure, Blood pressure change, Number of strokes	12 M	
Walma 1997 [31]	NL	Primary care	RCT	Overall202Disc102 Cont100	Disc76.1Cont76.1	Female151Male51	Disc147 Cont147	Disc81 Cont81	Furosemide, thiazide, triamterene monotherapy	receiving diuretics for at least 6 months and had noovert heart failure or hypertension	History of acute heart failurefurosemide over 80 mg/daymean of three BP values >180/100 mmHghypercalciurianephrotic syndromeglaucomafixed combination of diuretics with beta blockers or ACEIbeta blockers, diuretics and vasodilators for hypertensionuse of diuretic for which no placebo was available	Successful withdrawal of diuretics, congestive heart failure, Blood pressure change	6 M	
Moonen 2015 [32]	NL	Primary Care	RCT	Overall356 Disc180 Cont176	Disc81.1Cont81.5	Female209Male 147	Disc148.8Cont147	Disc82.3Cont80.0	Beta-Blocker, Diuretics, Angiotensin-converting enzyme inhibitors, Angiotensin receptor blocker; Calcium channel blocker	Discontinuation of antihypertensive treatment	Patients 75 years or olderactive antihypertensive treatmentsystolic BP (SBP) of 160 mm Hg or lessMini-Mental State Examination (MMSE) score of 21 to 27	Dementiaantihypertensives for reasons other than hypertensionangina pectoriscardiac arrhythmiaheart failure, myocardial infarction or a coronary reperfusion procedurehistory of stroke or transient ischemic attackhistory of peripheral arterial disease, myocardial infarction, or a coronary reperfusion procedurepersons with diabetes mellitus could participate if their SBP was 140 mm Hg or less	Overall cognitive compound scorechanges in psychological and general daily functioningChange (from baseline) in diastolic and systolic blood pressureNumber of deathsNumber of myocardial infarctionsNumber of transient ischemic attacksNumber of strokesNumber of hospitalizations	16 W
Nijst 2020 [34]	BE	Hospital	RCT	Overall80 Disc60 Cont20	70	Female41 Male39	Disc (RA)121Disc (BB)118Disc (RA, BB)132Cont 130	Disc (RA) 73Disc (BB)67Disc (RA, BB)72Cont71	Angiotensin-converting enzyme inhibitors, Angiotensin receptor blockers, beta-blockers, mineralocorticoid receptor antagonists, loop diuretics, ivabradine, other	Discontinuation of RAAS Inhibitor, beta-blocker or both	≥18 and able to give informed consent6 months CRT treatment prior inclusionLVEF ≥50%and normal LV volumeno hospital admission for worsening heart failure signs or symptoms within the past 6 monthsoptimal pharmacological therapy according to guideline recommendationsclinical euvolemic stateNYHA I or II	Ischemic cardiomyopathy with evidence of scarring or akinesiaand/or thin-walled myocardium in 2 LV wall segments on echocardiography before CRT device implantation known severe coronary atherosclerosis (stenosis - ≥80%)uncontrolled hypertension valvular heart disease of moderate or greater severityany condition with expected survival of less than 2 years in good functional status history of sustained ventricular arrhythmia and/or sustained episodes of atrial tachyarrhythmia detected by the CRT device after normalization of LVEF and cardiac dimensionsknown diabetic nephropathy renal dysfunction with proteinuria	Recurrence of negative remodelling, composite safety endpoint of all-cause mortality, heart-failure related hospitalizations, incidence of sustained ventricular arrhythmias at 24 month	24 M
Bogaerts 2024 [35]	NL	Long term Care	RCT	Overall205 Disc101 Cont104	Disc85.3Cont86.6	Female163 Male42	Disc134Cont 133	Overall Disc72Cont73	Angiotensin converting enzyme (ACE) inhibitor, angiotensin-II-receptor blocker, beta blocker, calcium antagonist or diuretic	Discontinuation of antihypertensive drugs prescribed for the indication of hypertension	Moderate-to-severe dementia according to the Reisberg Global Deterioration Scale (score 5, 6 or 7), hypertension and SBP ≤160 mmHg	estimated life expectancy of less than 4 months; heart failure class III–IV according to the functional classification of the New York Heart Association; current angina pectoris; or a recent myocardial infarction, stroke or coronary reperfusion procedure	change from baseline in neuropsychiatric symptoms and QoL, care dependency, functional status, care-related QoL of (non-professional) caregivers, cognitive status, apathy symptoms presence of delirium, number of deaths, transient ischaemic attacks, strokes, myocardial infarctions, hospitalizations	16 W
Sheppard 2024 [24]	UK	Primary Care	RCT	Overall564 Disc280 Cont284	Overall84.8	Female273 Male291	Disc129.4Cont 130.5	Disc68.4Cont 70.1	ACE-Inhibitors, Angiotensin II receptor blocker, Calcium channel blocker, B-blocker, Thiazide and related diuretics	Discontinuation of one antihypertensive drug	Same as Sheppard 2020	Same as Sheppard 2020	All-cause hospitalisation or Mortality, number of strokes, number of myocardial infarctions	48 M

*Abbreviations*: *ACEI* Angiotensin Converting enzyme Inhibitors, *BB* Beta-blockers, *BE* Belgium, *BL* baseline, *BP* blood pressure, *CA* Canda, *cont* continuation, *CRT* cardiac resynchronization therapy, *disc* discontinuation, *FUP* Follow up, *Ger* geriatric, *Inst* institution, *LV* Left ventricular, *LVEF* Left Ventricular Ejection Fraction, *M* months, *N* number, *NL* the Netherlands, *NYHA* New York Heart Association, *RA* RAAS inhibitors, *RCT* randomized controlled trials, *UK* United Kingdom, *W* weeks

**Table 2:** Characteristics of observational studies

AuthorYear	Country	Setting	Study Design	*N*	Age(Years)	Sex	AntihypertensiveTreatment	Inclusion criteria	Exclusion Criteria	Outcomes	FUP
Teichert 2007 [29]	NL	General practices, hospitals	Nested case control studies	Overall2588Cases MI148Controls2440	Cases72Controls69	Female1588Male1000	Beta-Blocker	≥55 years subjects who had used a beta-blocker for atleast 30 days during the study period 1.1.1991-1.1.2002,patients who had filled any prescription at the community pharmacy within 90 days prior to the index dates in the cohort	None	Incident myocardial infarction, death	30 D – 180 D
Song 2018 [25]	USA	Mix of inpatient, outpatient and data from nursing homes or purchased	Cohort Study	Overall2212Disc239Cont1973	80.5	Male97.7%	Thiazide diuretics, calcium channel blocker, angiotensin converting enzyme inhibitor, angiotensin receptor blocker, beta blocker	long stay residents treated for hypertension≥ 65 years	falls associated with hospital admission as index event	Recurrent fall, mortality, hospital admissions	30 D
van Dalen 2019 [26]	NL	Primary Care	Prospectiveobservational cohort study (using the data from the Prevention of Dementia by Intensive Vascular care perDIVA trial)	Overall1541Disc85Cont1451	Disc74.5Cont74.3	Female828Male623		aged 70-78 at baseline,participants using antihypertensive drugs at baseline	dementia or disorders likely to impede successful long-term follow-up according to the GP (terminal illness, alcoholism)	Dementia, dementia or mortality, mortality	6-8 Y
Qiao 2020 [36]	USA	Primary care	Retrospective,propensity score-matched cohort study	Overall3909Disc2674Cont1235	Disc73Cont74	Female2406Male603	Angiotensin converting enzyme inhibitor, angiotensin receptor blocker	individuals initiated ACE-I or ARB therapy between Jan 1, 2004-Dec 31, 2018Outpatient eGFR decreased to 30 ml/min after therapy initiation	individuals stoppedtreatment,restarting treatment within 6 monthsdeath before follow-upor prevalent ESKD at baseline	All-cause mortality, major cardiovasc events, end-stage kidney disease	5 Y
Fu 2021 [28]	SE	Nephrologist care	Cohort study (Swedish RenalRegistry [SRR] 2007-2017)	Overall10'254 Disc1311Cont8484	Disc74Cont71	Female3526Male6728	Renin-angiotensin system inhibitors (RASi)	≥18 yearregistered in the SRR afterJanuary 1, 2007new CKD G4 (whose eGFR decreased to <30 ml/min) had taken RASi for more than 80% of the 2 years before the date (needed to be adherent)	not adherent to thetreatmenthistory of kidney transplantationpatients with missing BP measurements at the time of eGFR to <30 ml/min or those that stopped RASi before the decrease in eGFR	All-cause mortality, major cardiovasc events, kidney replacement treatment	5 Y
Aubert2021 [37]	USA	US primary care Veterans Health Administration healthcare system	Cohort Study	Overall228753Disc72,672	Overall75.2	Male98.2%		All patients aged ≥ 65 with primary care at VHA, hypertension and tightly controlled BP (SBP < 130 mmHg) with one or more antihypertensive on 2 or more visits	Death before end of follow- up	Any hospitalization or emergency department visit for acute cardiovascular events, syncope, fall injury	9 M
Hasegawa 2022 [33]	JP	Frailty clinic	Cohort study	Overall78Disc19Cont59	Overall77	Female54Male24	Antihypertensive drugs, vasodilators, thiazide derivates and preparations	Consecutive patients attending the frailty clinic at the National centre for Geriatrics and Gerontology, receiving antihypertensive drugs at their first visit and who attended a follow-up visit	No antihypertensive treatment, loss of follow up after the first visit and up to 1 year	Change in Kihon checklist score and physical performance	1 Y

*Abbreviations*: *ACE-I* Angiotensin Converting Enzyme Inhibitors, *ARB* Angiotensin Receptor Blockers, *BP* blood pression, cardiovasc, cardiovascular, cont continuation, *CKD* chronic kidney disease, *D* days, disc discontinuation, *eGFR* estimated glomerular filtration rate, *ESKD* end stage kidney disease, *FUP* Follow up, *JP* Japan, *M* months, *MI* myocardial infarction, *NL* the Netherlands, *N* number, *RASi* Renin Angiotensin System Inhibitors, *SBP* Systolic blood pressure, SE, Sweden, *SRR* Swedish Renal Register, *VHA* Veterans Health Administration, *Y* years

### Risk of bias

**Fig. 2:**
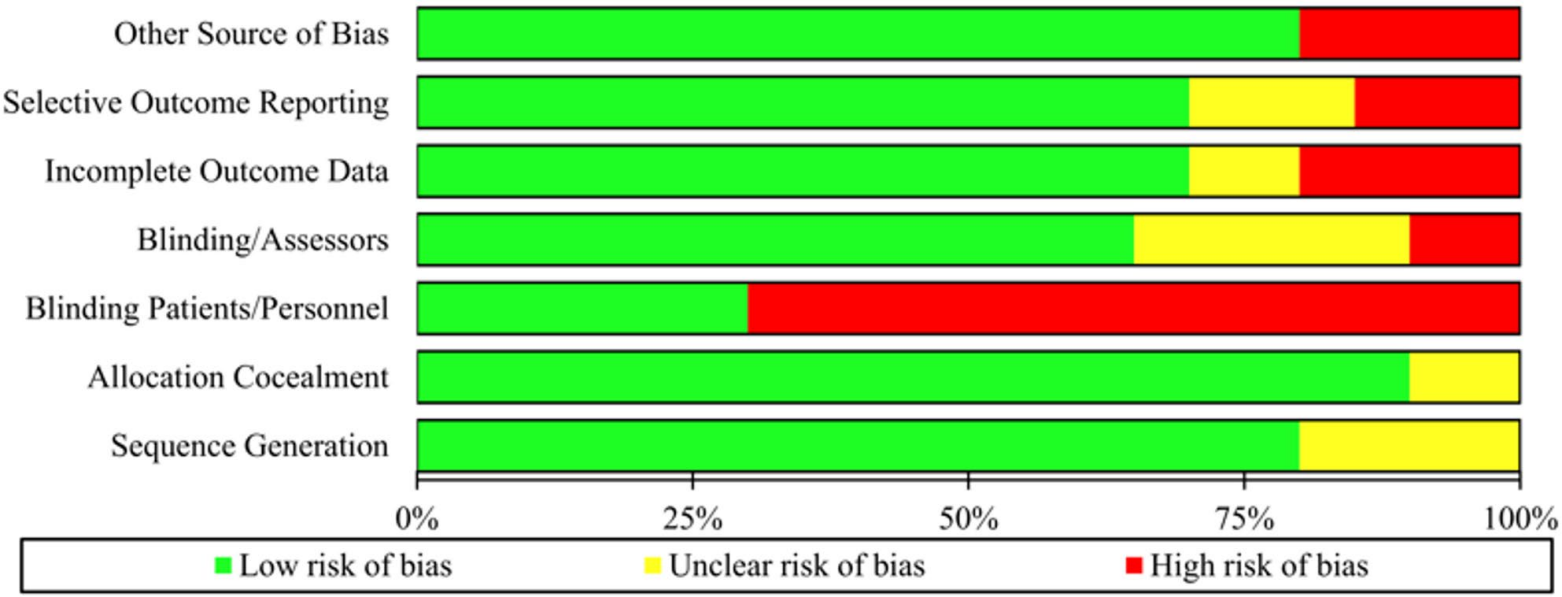
Risk of bias graph: Authors’ judgments about each risk of bias reported as percentages across all included studies

**Fig. 3:**
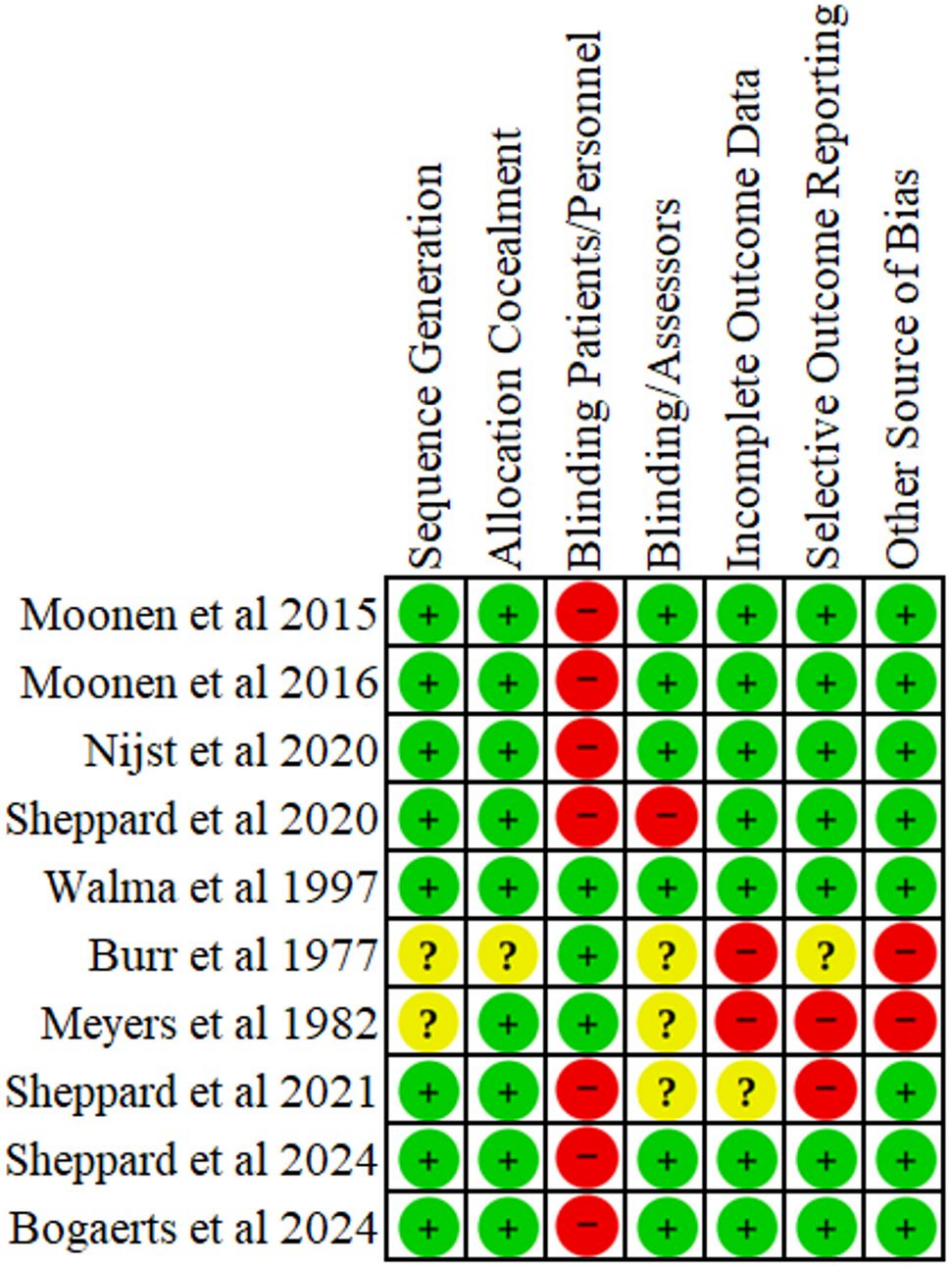
Risk of Bias Summary. Review authors' judgements about each risk of bias item for the included studies. Green is for low risk, yellow for unclear risk and red for high risk of bias

The included RCTs differed in their risk of bias (Figs. 2 and 3). Random sequence generation was adequately described in five of seven RCTs. Only one RCT did not provide sufficient information and the risk of bias was considered as unclear [23]. Only one study did not report on allocation concealment [23]. The blinding of participants and personnel was described only in three RCTs. However, four studies lacked blinding and had high risk of bias [24, 32, 34, 35]. Information on blinding of outcome assessment was available in five studies; the remaining studies were at unclear risk of detection bias [23, 27]. Most studies showed low attrition rates due to incomplete outcome data, resulting in low risk of bias, with the exception of two studies with high dropout rates and inadequate handling of missing data [23, 27]. Most of the studies showed no evidence of selective reporting, though one study was at unclear risk [23] and one at high risk of bias [27].

With the exception of Hasegava et al. [33], the average methodological quality of the included cohort studies was rated as ‘good’ according to the Newcastle Ottawa Scale for cohort studies (Appendix Sect. 8). A table reporting which confounders the included observational studies were adjusted for is reported in the Appendix Sect. 9.

### All-cause mortality

#### RCTs

**Fig. 4:**
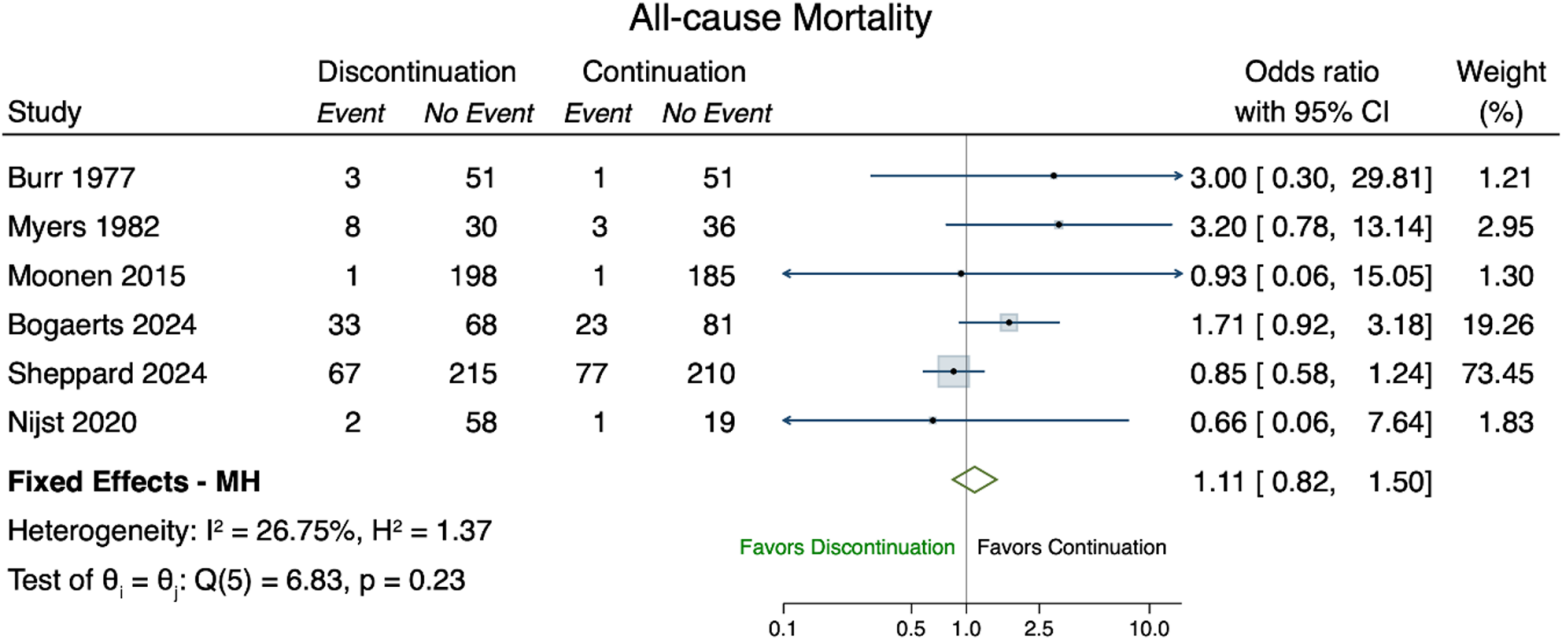
Forest Plot all-cause mortality. Forest plot of the odds ratios of all-cause mortality (event) comparing discontinuation (treatment) versus continuation (control). Fixed Effects - MH denotes the Mantel–Haenszel fixed effect odds ratio of the selected studies (green diamond). The I^2 and H^2 measure the level of levels of inconsistency and heterogeneity (full homogeneity: H^2=1; negligible inconsistency: I^2∈[0,40])

Six RCTs reported (all-cause) mortality [23, 24, 27, 32, 34, 35]. With a total of 1,590 participants, the pooled OR for all-cause mortality after deprescribing antihypertensives was 1.11 (95% CI 0.82–1.50) compared to continuation (Fig. 4) and did not change when considering the duration of follow-up in a subgroup analysis (Appendix Sect. 5).

#### Observational studies

The cohort studies of Qiao et al. [36] and Fu et al. [28] provided evidence of an increased 5-year mortality risk following discontinuation of ACE-I/ARB. Qiao et al. [36] found that 434 of the 1,235 individuals (35.1%) who discontinued therapy after progression of chronic kidney disease died within five years, compared to 786 of the 2,674 individuals (29.4%) who continued therapy. This association between deprescribing of ACE-I/ARB and higher mortality risk persisted after adjustment for baseline covariates with a Hazard Ratio [HR] of 1.39 (95% CI 1.20–1.60). Similarly, Fu et al. [28] found a higher the 5-year mortality risk among those who discontinued ACE-I/ARB therapy (54.5%, 95% CI 48.5%-61.2% ) compared to those who continued treatment ( 40.9%, 95% CI 39.0%-42.8%), yielding an absolute risk difference of 13.6 (95% CI 7.0-20.3). In the study by Song et al. [25], deprescribing antihypertensive medications was associated with a higher probability of death among participants with systolic blood pressure (SBP) between 101 and 120 mmHg, but not among those with SBP between 80 and 100 mmHg (adjusted marginal effect (AME) 2.1%; p-value = 0.07). In van Dalen et al. [26] the hazard for mortality was 16–21% higher following discontinuation of antihypertensive medications, although the results were not statistically significant (all p -≥ 0.38). Aubert et al. [37] found that participants who underwent deprescribing of antihypertensive treatment, either through dose reduction or a reduction in the number of medications, had the highest mortality rate of 4.1%, compared to those with treatment intensification (3.2%), and those who continued their treatment unchanged (1.8%, *p* < 0.001).

### Myocardial infarction

#### RCTs

**Fig. 5:**
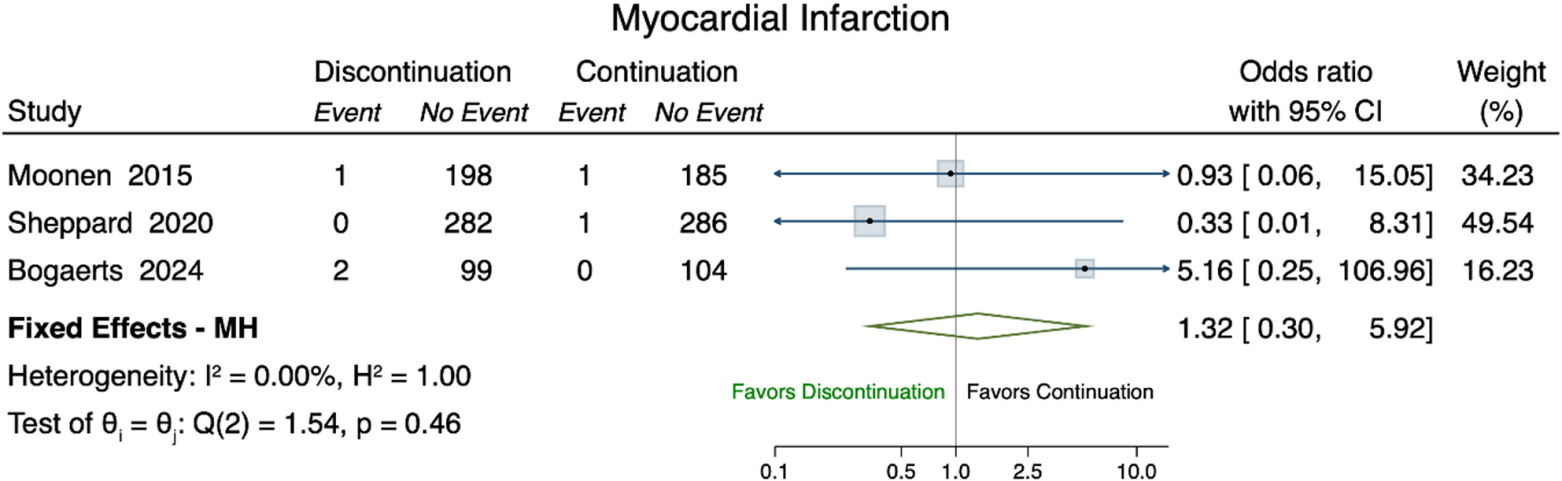
Forest Plot myocardial infarction. Forest plot of the odds ratios of myocardial infraction (event) comparing discontinuation (treatment) versus continuation (control). Overall represents odds ratio denotes the MH fixed effect odds ratio of the selected studies. The I^2 and H^2 measure the level of levels of inconsistency and heterogeneity (full homogeneity: H^2=1; negligible inconsistency: I^2∈[0,40])

Three studies reporting on myocardial infarction could be included in a meta-analysis [30, 32, 35] **(**Fig. 5**)**. The pooled OR was estimated at 1.32 (95% CI 0.30–5.92).

#### Observational studies

Among the observational studies, only Teichert et al. [29] specifically reported myocardial infarction as a primary endpoint. They found an increased risk after discontinuation of selective beta-blockers, with a risk ratio (RR) 2.70 (95% CI 1.06–6.89) within the first 30 days, and a RR of 2.44 (95% CI 1.07–5.59) between 30 and 180 days after treatment discontinuation.

### Heart failure

**Fig. 6:**
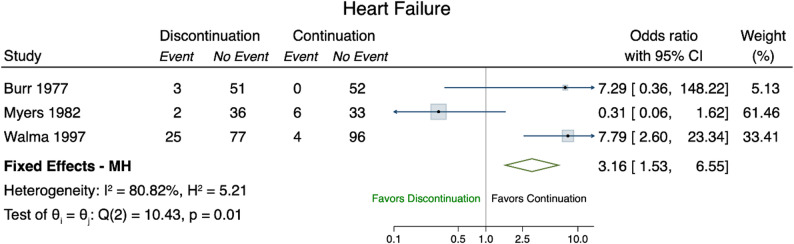
Forest plot heart failure. Forest plot of the odds ratios of heart failure (event) comparing discontinuation (treatment) versus continuation (control). Fixed Effects - MH denotes the Mantel–Haenszel fixed effect odds ratio of the selected studies (green diamond). The I^2 and H^2 measure the level of levels of inconsistency and heterogeneity (full homogeneity: H^2=1; negligible inconsistency: I^2∈[0,40])

Three RCTs reported on heart failure as an outcome [23, 27, 31]. Our meta-analysis showed that the odds of heart failure among those who discontinued diuretics were 3.16 (95% CI 1.53–6.55) times higher compared to those who continued therapy (Fig. 6).

### Stroke

#### RCTs

**Fig. 7:**
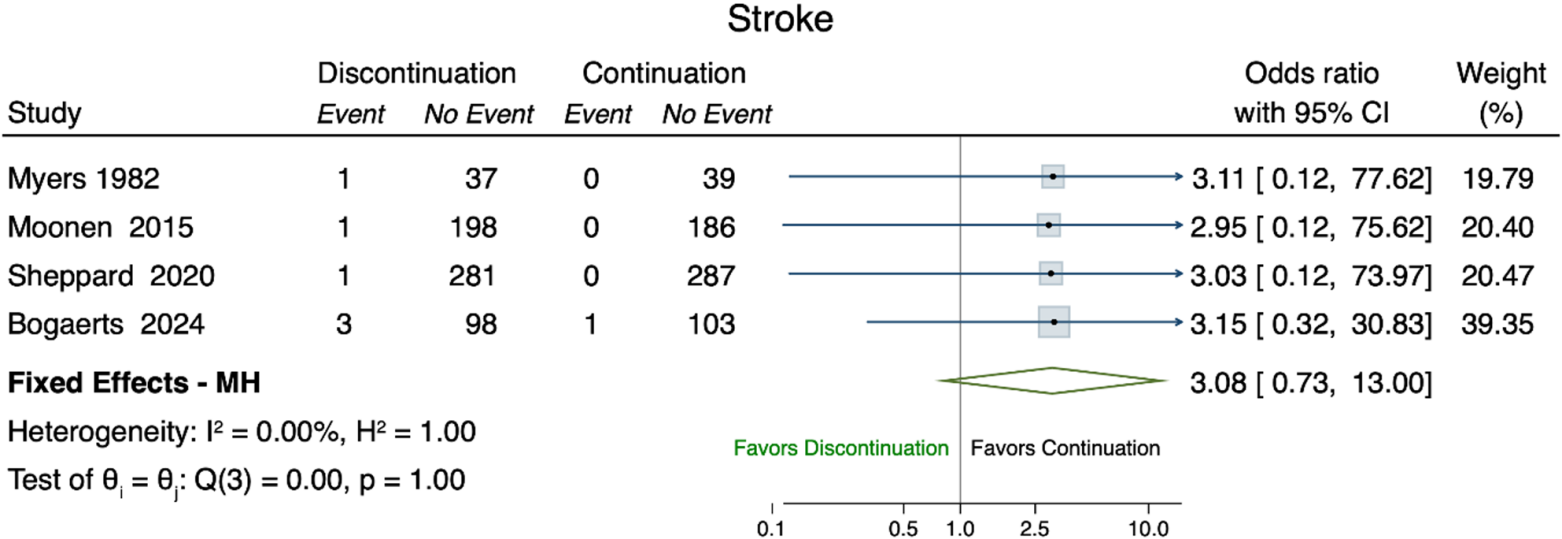
Forest plot stroke. Forest plot of the odds ratios of stroke (event) comparing discontinuation (treatment) versus continuation (control). Fixed Effects - MH denotes the Mantel–Haenszel fixed effect odds ratio of the selected studies (green diamond). The I^2 and H^2 measure the level of levels of inconsistency and heterogeneity (full homogeneity: H^2=1; negligible inconsistency: I^2∈[0,40])

Four studies were included in the meta-analysis (Fig. 7) [27, 30, 35, 38], which encompassed a total of 1,410 patients. The pooled analysis demonstrated a higher risk of stroke, yet not statistically significant, in the discontinuation group, with a pooled OR of 3.08 (95% CI 0.73-13.00).

#### Observational studies

Song et al. [25] found no association between antihypertensive deintensification and hospitalizations due to vascular events (e.g., stroke or myocardial infarction). No observational studies specifically reporting stroke as an outcome were identified.

### Major Cardiovascular Events (MACE)

The only RCT reporting on MACE was the long-term follow-up of the OPTIMISE trial [24]. In this study MACE was defined as hospital admission for non-fatal stroke, myocardial infarction, heart failure, or cardiovascular mortality. There was no significant difference between groups, with an adjusted HR of 1⋅00 (95% CI 0⋅68–1.46).

#### Observational studies

Fu et al. [28] reported on the 5-year risk of MACE, defined as a composite outcome of mortality, myocardial infarction, and cerebrovascular events, in patients with advanced chronic kidney disease. The incidence rate of MACE was 47.6% (95% CI 45.9%-49.4%) in the group continuing RAAS inhibitors compared to 59.5% (95% CI 53.8%-66.1%) in the group that discontinued RAAS inhibitors treatment.

Qiao et al. [36] found that patients with an estimated glomerular filtration rate of < 30 ml/min/1.73 m^2^ who discontinued ACE-I or ARB had a significantly higher risk of MACE (defined as death, myocardial infarction, percutaneous coronary intervention, or coronary artery bypass) compared to those who continued treatment (HR 1.37; 95% CI 1.20–1.56).

Similarly, Aubert et al. [37] reported a higher adjusted absolute risk of cardiovascular events in participants who reduced the dose of antihypertensives medications (12.3%; 95% CI 12.0%-12.5%) compared to those maintained the same dose (9.1%; 95%CI 8.9%-9.2%).

### Orthostatic hypotension

Orthostatic hypotension was reported in the study by Moonen et al. [38]. In the intention-to-treat analysis, participants in the discontinuation group had a 1.31-fold (95%CI 0.92–1.87) higher probability of recovery from orthostatic hypotension compared with the control group.

In the study by Hasegawa et al. [33] orthostatic hypotension was observed in 50–65% of older participants taking antihypertensive medications. However, the study did not evaluate the effect of discontinuation of antihypertensive medications on orthostatic hypotension.

### Falls

#### RTCs

In the OPTIMISE trial [30], there were 2 falls (0.7%) in the medication reduction group compared to 1 fall (0.35%) in the control group.

In the study of Bogaerts et al. [35], discontinuation of antihypertensive medications was associated with a higher risk of falling (adjusted mean ratio 2.21, 95%CI 1.56–3.13).

#### Observational studies

In the study by Song et al. [25], reduction of antihypertensive medications was associated with a lower risk of falls among participants with low systolic blood pressure (80–100 mmHg), with a weighted marginal effect (WME) of -13.6% (*p* < 0.01).

Aubert et al. [37] reported that deintensification of treatment was associated with a 1.5% (95% CI 1.1%-1.9%) greater risk of fall-related injury compared with continued treatment. However, the lowest fall risk was observed in the deintensification group among participants with a low baseline SBP (< 95 mmHg).

Hasegawa et al. [33] found that the fall risk score decreased in in the continuation group compared to the discontinuation group after 1 year follow-up among participants attending a frailty clinic.

## Discussion

This systematic-review and meta-analysis summarized available evidence on deprescribing antihypertensive drugs in older adults. Our findings highlight the lack of high-quality studies assessing the feasibility and safety of deprescribing antihypertensive medications, with many included studies exhibiting notable limitations. Overall, evidence suggests that antihypertensive deprescribing should be approached with caution. Given the notable clinical heterogeneity among older adults deprescribing antihypertensives is not universally appropriate and should instead be individualized taking into account possible benefits, risks, and patient preferences. The estimated effects on mortality indicate a potential increased risk associated with discontinuation, although the confidence intervals were wide due to the small number of studies and low event rates.

Event rates for myocardial infarction and stroke were very low, making these outcomes difficult to interpret with confidence. The low event rates of both myocardial infarction and stroke hinder the interpretation of these findings with confidence. In observational studies, discontinuation of antihypertensive medications (specifically RAAS inhibitors, ACE-I, ARB) was associated with an increased 5-year mortality risk and MACE in individuals with advanced chronic kidney disease. However, event rates for specific cardiovascular outcomes, such as myocardial infarction, stroke and heart failure were low, resulting in wide 95% CIs around pooled estimates. The observed association between antihypertensive deprescribing and worse outcomes may, in part, be explained by a higher baseline risk of the included populations [29]. Additionally, it is possible that that discontinuation of treatment revealed or accelerated the progression of underlying conditions [37]. In contrast to these findings, the long-term results of the OPTIMISE trial provide weak evidence that deprescribing antihypertensive medications may be safe in older, community-dwelling individuals with well-controlled blood pressure on two or more antihypertensive agents [24]. One possible explanation is that long-term antihypertensive treatment may induce favorable structural and functional vascular changes, potentially sustaining blood pressure control even after medication reduction. The DANTON study [35]aimed to investigate whether deprescribing antihypertensive medication in nursing home residents in the Netherlands could reduce neuropsychiatric symptoms and improve quality of life. However, after two years of recruitment, fewer than half of the planned participants had been enrolled, and 63 serious adverse events had occurred among participants; as a result, the trial was terminated early for futility.

To what extent contextual differences across community, hospital, and long-term care settings influence the effects of antihypertensives deprescribing remains unclear and needs further investigation.

Our review builds on the Cochrane review by Reeve et al. [10], which was the first systematic review and meta-analysis to investigate the feasibility and safety of withdrawing antihypertensive medications across multiple outcomes, including mortality, cardiovascular risk, blood pressure control, and quality of life. Notably, the Cochrane review reported an increase in systolic blood pressure (SBP) and diastolic blood pressure (DBP) following withdrawal of antihypertensive medications, but found no evidence of difference in mortality [10]. This conclusion could be confirmed by un update of this review 2025 [11]. A recent systematic review on deprescribing diuretics concluded that discontinuation may be safe and feasible for selected participants, although the overall quality of evidence was low [12]. Deprescribing was generally well accepted in participants using diuretics for hypertension. However, those using diuretics for heart failure appeared to have higher risk of developing peripheral oedema following discontinuation of diuretics [12], which was associated with a greater likelihood of resuming diuretics therapy [31].

This review has several limitations. We acknowledge the last literature search was conducted in July 2024. While this represents a structural limitation inherent to current systematic review workflows, the recently published update by Reeve. E et al. 2025 [11] did not identify any additional studies beyond those included in 2020. Consequently, the findings of this review remain consistent. Although switching to a different class of antihypertensive medication is sometimes a pragmatic form of de-intensification in real-world care, switching medication was not considered as deprescribing. Our definition of deprescribing was limited to dose reduction or discontinuation to maintain internal consistency across studies.

With fewer than ten studies per outcome we could not perform regression-based bias tests, because these tests have low statistical power and may lead to misleading conclusions. Event rates for key cardiovascular outcomes, such as myocardial infarction and strokes, were very low, hindering the interpretation of the findings. Furthermore, data on other clinically relevant outcomes such as falls, orthostatic hypotension and other adverse effects of antihypertensive medications, were limited. Although sex-specific differences may be important in interpreting the results, we were unable to perform subgroup analysis by gender, as the data were not reported separately. Our search was restricted to original studies published in English, possibly missing relevant studies published in other languages. The generalizability of our findings to the broader older adult population is limited. Many older individuals in routine clinical practice are characterized by multimorbidity and frailty [39], yet such populations are often excluded or underrepresented in clinical trials. This highlights a significant gap in the current evidence base. Because available data are insufficient for statistical subgroup meta-analysis on frailty or comorbidity, we could only report narratively on differences by frailty or comorbidity.

This review focuses specifically on older adults (65 years and older), enhancing the applicability and generalizability of the findings to this population. In contrast to the Cochrane review of Reeve et al. [10], which included only six RCTs, four of which examined the discontinuation of diuretics, our review also incorporated RCTs investigating the discontinuation of non-diuretic antihypertensive agents. This broader scope allows for a more comprehensive evaluation of deprescribing across a wider range of antihypertensive drug classes, enabling more nuanced considerations of their potential benefits and harms.

Furthermore, we also included observational studies, which is a notable strength. Unlike RCTs, observational studies do not typically exclude participants based on health status or frailty and therefore are likely to better reflect the real-world population of older adults.

Translating our findings into straightforward recommendations for routine clinical practice is challenging, as decisions to withdraw antihypertensive medications must be made within the framework of shared decision-making, taking into consideration the patient’s clinical condition, individual preferences, and wishes/values.

## Conclusion

Overall, the effects of deprescribing antihypertensive medications remains unclear. While it appears to be a feasible option for selected patients, particularly those with well-controlled blood pressure in outpatient-settings, there is lack of significant evidence on outcomes that are especially relevant to older adults, such as functional status and quality of life. This highlights the importance of individualized decision-making when considering deprescribing antihypertensive medications in this population. To support the safe and effective implementation of antihypertensive deprescribing in everyday clinical practice, further research is needed, particularly in populations characterized by frailty and multimorbidity, to clarify potential risks and benefits. 

## Supplementary Information

Supplementary Material 1.

Supplementary Material 2.

Supplementary Material 3.

## Data Availability

Data supporting the findings of this study were derived from resources available in the public domain. The authors confirm that the data are available within the article and/or its supplementary materials. Any additional data is available from the corresponding author upon request.
