# Supplementary material for: Deprescribing antihypertensive medications in older people: a systematic review and a meta-analysis
Source: BMC Geriatr. 2026 Jan 5;26:222. doi: 10.1186/s12877-025-06941-2 (PMC12911232; doi:10.1186/s12877-025-06941-2)
Supplement: Supplementary file 1 — Supplementary Material 1. [file 12877_2025_6941_MOESM1_ESM.docx]

**Appendix**

1. **Deviations from registered protocol in PROSPERO**

- **Review Titel and Basic Details**

No deviations from registered protocol in PROSPERO

- **Review objectives**

No deviations from registered protocol in PROSPERO. The two additional reviews on harms of continuing antihypertensives in the same study population and patients' preferences and values towards anthypertensives are currently under revision

- **Searching and Screening**

No deviations from registered protocol in PROSPERO

- **Eligibility Criteria**

No deviations from registered protocol in PROSPERO

- **Outcomes**

Main outcome: mortality, no deviations from registered protocol in PROSPERO

Secondary outcomes: due to missing or inconsistent reporting of certain secondary outcomes, we limited the secondary outcomes to myocardial infarction, heart failure, stroke, Major Adverse Cardiovascular Events (MACE), orthostatic hypotension, falls. Originally reported in PROSPERO protocol were:  blood pressure (BP; systolic and diastolic BP before and after deprescribing antihypertensive medications, including mean arterial pressure), myocardial infarction (fatal and non-fatal), stroke (fatal and non-fatal, ischaemic and haemorrhagic, transient ischaemic attack), hospitalizations (all-cause hospitalization, cardiovascular hospitalization, heart failure hospitalization), quality of life (QoL; measured using validated instruments, EQ5D), cognitive decline (measured using validated instruments, MOCA), orthostatic hypotension (postural hypotension), falls, side effects (typical side effects of antihypertensive treatment, for example ankle oedema, cough, and dizziness), need to restart medication within follow-up period, adverse drug reactions, and adverse drug withdrawal reactions.

- **Data Collection Process**

Data extraction: No deviations from registered protocol in PROSPERO

Risk of bias assessment: For randomized control studies we assessed the risk of bias in Covidence basing on the Cochrane Risk of Bias (Rob 2) tool, for observational studies we used the Newcastle Ottawa Scale, which is a deviation from registered protocol in PROSPERO (originally the use of the ROBINS-I tool was planned)

- **Planned Data Synthesis**

All analyses were carried out using STATA (version 18.0), using the commands in the ‘meta’ environment (StataCorp. 2023. Stata Statistical Software: Release 18. College Station, TX: StataCorp LLC).

Due to missining data we could not perform the originally planned subgroupanalysis based on gender, age, frailty, cognitive impairment, EoL; sensitivity analyses will be performed by age group, physical functioning and frailty.

1. **Full search strategies**

Database: Ovid MEDLINE(R) ALL (1946 -- 01/07/2024)

Search date: 02/07/2024

Search Strategy:

----------------------------------------------------------------------

ID Search Hits

-----------------------------

1 exp aged/ 3529878

2 (advanced years or ageing or aging or elder$ or elderly or frail or geriatric? or gerontolog$ or later life or nursing care or nursing home? or old age or oldest old or pensioner? or post-menopausal or postmenopausal or senior or seniors or aged or septuagenarian$ or octogenarian$ or nonagenarian$ or centenarian$ or supercentenarian$ or senium).mp. 6582150

3 (old$ adj3 (adult? or female? or male? or men or people or person or women or patient?)).tw,kf. 646699

4 ((over or old$) adj2 ("65" or "66" or "67" or "68" or "69" or "70" or "71" or "72" or "73" or "74" or "75" or "76" or "77" or "78" or "79" or "80" or "81" or "82" or "83" or "84" or "85" or "86" or "87" or "88" or "89" or "90" or "91" or "92" or "93" or "94" or "95" or "96" or "97" or "98" or "99" or "100") adj year?).tw. 36821

5 (aged or aging or ageing or elder$ or geriatric$ or gerontolog$).jw,nw. 217144

6 or/1-5 6884684

7 hypertension/ 264294

8 essential hypertension/ 2832

9 hypertens$.tw,kf. 537851

10 ((elevat$ or high$ or raised) adj2 (bp or blood pressure)).tw,kf. 52123

11 or/7-10 611170

12 exp antihypertensive agents/ 273260

13 exp Diuretics/ 84623

14 acetazolamide/ or amiloride/ or bendroflumethiazide/ or bumetanide/ or chlorothiazide/ or chlorthalidone/ or clopamide/ or cyclopenthiazide/ or ethacrynic acid/ or ethoxzolamide/ or furosemide/ or hydrochlorothiazide/ or hydroflumethiazide/ or indapamide/ or mefruside/ or methyclothiazide/ or metolazone/ or polythiazide/ or spironolactone/ or torsemide/ or triamterene/ or trichlormethiazide/ or xipamide/ 47283

15 (Acetazolamide or azosemide or buthiazide or Canrenone or Chlorthalidone or cicletanine or Clopamide or Cyclopenthiazide or cyclothiazide or drospirenone or Eplerenone or Ethacrynic Acid or ethanolamine O-sulfate or Ethoxzolamide or ethylisopropylamiloride or etozolin or fenquizone or indacrinone or Indapamide or Isosorbide or Mannitol or Mefruside or Metolazone or "N-(3-(1-cyclopropyl-1-(2,4-difluorophenyl)ethyl)-1H-indol-7-yl)methanesulfonamide" or "N-4,4-dimethyl-2-thioxo-1,4-dihydro-2H-3,1-benzoxazin-6-ylthiophene-2-sulfonamide" or piretanide or quinethazone or Spironolactone or Torsemide or Triamterene or tripamide or Xipamide).tw,kf. 49217

16 exp thiazides/ 15966

17 (antihypertens$ or hypotensiv$).tw,kf. 78750

18 ((ceiling or loop) adj diuretic?).tw,kf. 3896

19 (amiloride or benzothiadiazine or bendroflumethiazide or bumetanide or chlorothiazide or cyclopenthiazide or furosemide or hydrochlorothiazide or hydroflumethiazide or methyclothiazide or metolazone or polythiazide or trichlormethiazide or veratide or thiazide?).tw,kf. 39278

20 (chlorthalidone or chlortalidone or phthalamudine or chlorphthalidolone or oxodoline or thalitone or hygroton or indapamide or metindamide).tw,kf. 2835

21 or/12-20 408071

22 exp angiotensin-converting enzyme inhibitors/ 48154

23 angiotensin converting enzyme inhibit$.tw,kf. 23842

24 (ace adj2 inhibit$).tw,kf. 22198

25 acei.tw,kf. 5234

26 (alacepril or altiopril or ancovenin or benazepril or captopril or ceranapril or ceronapril or cilazapril or deacetylalacepril or delapril or derapril or enalapril or epicaptopril or fasidotril or fosinopril or foroxymithine or gemopatrilat or idapril or imidapril or indolapril or libenzapril or lisinopril or moexipril or moveltipril or omapatrilat or pentopril$ or perindopril$ or pivopril or quinapril$ or ramipril$ or rentiapril or saralasin or s nitrosocaptopril or spirapril$ or temocapril$ or teprotide or trandolapril$ or utibapril$ or zabicipril$ or zofenopril$ or Aceon or Accupril or Altace or Capoten or Lotensin or Mavik or Monopril or Prinivil or Univas or Vasotec or Zestril).tw,kf. 29250

27 or/22-26 71340

28 exp Angiotensin Receptor Antagonists/ 28715

29 (angiotensin adj3 receptor antagon$).tw,kf. 4051

30 (angiotensin adj3 receptor block$).tw,kf. 14507

31 (arb or arbs).tw,kf. 9797

32 (abitesartan or azilsartan or candesartan or elisartan or embusartan or eprosartan or forasartan or irbesartan or losartan or milfasartan or olmesartan or saprisartan or tasosartan or telmisartan or valsartan or zolasartan or Atacand or Avapro or Benicar or Cozaar or Diovan or Micardis or Teveten).tw,kf. 22294

33 or/28-32 44546

34 exp calcium channel blockers/ 91917

35 (amlodipine or aranidipine or barnidipine or bencyclane or benidipine or bepridil or cilnidipine or cinnarizine or clentiazem or darodipine or diltiazem or efonidipine or elgodipine or etafenone or fantofarone or felodipine or fendiline or flunarizine or gallopamil or isradipine or lacidipine or lercanidipine or lidoflazine or lomerizine or manidipine or mibefradil or nicardipine or nifedipine or niguldipine or nilvadipine or nimodipine or nisoldipine or nitrendipine or perhexiline or prenylamine or semotiadil or terodiline or tiapamil or verapamil or Cardizem CD or Dilacor XR or Tiazac or Cardizem Calan or Isoptin or Calan SR or Isoptin SR Coer or Covera HS or Verelan PM).tw,kf. 67400

36 (calcium adj2 (antagonist? or block$ or inhibit$)).tw,kf. 43113

37 or/34-36 128133

38 (methyldopa or alphamethyldopa or amodopa or dopamet or dopegyt or dopegit or dopegite or emdopa or hyperpax or hyperpaxa or methylpropionic acid or dopergit or meldopa or methyldopate or medopa or medomet or sembrina or aldomet or aldometil or aldomin or hydopa or methyldihydroxyphenylalanine or methyl dopa or mulfasin or presinol or presolisin or sedometil or sembrina or taquinil or dihydroxyphenylalanine or methylphenylalanine or methylalanine or alpha methyl dopa).mp. 16623

39 (reserpine or serpentina or rauwolfia or serpasil).mp. 20773

40 (clonidine or adesipress or arkamin or caprysin or catapres$ or catasan or chlofazolin or chlophazolin or clinidine or clofelin$ or clofenil or clomidine or clondine or clonistada or clonnirit or clophelin$ or dichlorophenylaminoimidazoline or dixarit or duraclon or gemiton or haemiton or hemiton or imidazoline or isoglaucon or klofelin or klofenil or m-5041t or normopresan or paracefan or st-155 or st 155 or tesno timelets).mp. 21612

41 exp hydralazine/ 4780

42 (hydralazin$ or hydrallazin$ or hydralizine or hydrazinophtalazine or hydrazinophthalazine or hydrazinophtalizine or dralzine or hydralacin or hydrolazine or hypophthalin or hypoftalin or hydrazinophthalazine or idralazina or 1-hydrazinophthalazine or apressin or nepresol or apressoline or apresoline or apresolin or alphapress or alazine or idralazina or lopress or plethorit or praeparat).tw,kf. 5060

43 or/38-42 62222

44 exp adrenergic beta-antagonists/ 87796

45 (acebutolol or adimolol or afurolol or alprenolol or amosulalol or arotinolol or atenolol or befunolol or betaxolol or bevantolol or bisoprolol or bopindolol or bornaprolol or brefonalol or bucindolol or bucumolol or bufetolol or bufuralol or bunitrolol or bunolol or bupranolol or butofilolol or butoxamine or carazolol or carteolol or carvedilol or celiprolol or cetamolol or chlortalidone cloranolol or cyanoiodopindolol or cyanopindolol or deacetylmetipranolol or diacetolol or dihydroalprenolol or dilevalol or epanolol or esmolol or exaprolol or falintolol or flestolol or flusoxolol or hydroxybenzylpinodolol or hydroxycarteolol or hydroxymetoprolol or indenolol or iodocyanopindolol or iodopindolol or iprocrolol or isoxaprolol or labetalol or landiolol or levobunolol or levomoprolol or medroxalol or mepindolol or methylthiopropranolol or metipranolol or metoprolol or moprolol or nadolol or oxprenolol or penbutolol or pindolol or nadolol or nebivolol or nifenalol or nipradilol or oxprenolol or pafenolol or pamatolol or penbutolol or pindolol or practolol or primidolol or prizidilol or procinolol or pronetalol or propranolol or proxodolol or ridazolol or salcardolol or soquinolol or sotalol or spirendolol or talinolol or tertatolol or tienoxolol or tilisolol or timolol or tolamolol or toliprolol or tribendilol or xibenolol).tw,kf. 69969

46 (beta adj2 (adrenergic? or antagonist? or block$ or receptor?)).tw,kf. 115691

47 or/44-46 179675

48 exp adrenergic alpha antagonists/ 54121

49 (alfuzosin or bunazosin or doxazosin or metazosin or neldazosin or prazosin or silodosin or tamsulosin or terazosin or tiodazosin or trimazosin).tw,kf. 15563

50 (adrenergic adj2 (alpha or antagonist?)).tw,kf. 21359

51 ((adrenergic or alpha or receptor?) adj2 block$).tw,kf. 69739

52 or/48-51 131152

53 Vasodilator Agents/ 44726

54 (vasoactive antagonist$ or vasodilator$ or vasorelaxant$).tw,kf. 42255

55 Ganglionic Blockers/ 2976

56 (ganglionic block$ or ganglioplegic agent$).tw,kf. 2212

57 or/53-56 78847

58 21 or 27 or 33 or 37 or 43 or 47 or 52 or 57 745081

59 Deprescriptions/ or (Drug Prescriptions/ and (19* or 200* or 2010* or 2011* or 2012* or 2013* or 2014* or 2015*).ed.) or Withholding Treatment/ 37009

60 (deprescri$ or de-prescri$).tw,kf. 2649

61 ((withhold$ or withheld or withdraw$ or discontinu$ or reduced or reduces or reducing or reduction? or ceased or ceasing or cessation? or stop$ or taper$) adj3 (antihypertens$ or anti-hypertens$ or diuretic? or dosage? or dose or doses or drug$ or medicat$ or prescri$ or treatment$ or therapy or therapies or therapeutics)).tw,kf. 301242

62 or/59-61 335474

63 randomized controlled trial.pt. 616258

64 controlled clinical trial.pt. 95564

65 randomi#ed.ab. 777022

66 placebo.ab. 249612

67 clinical trials as topic/ 202739

68 randomly.ab. 436634

69 trial.ti. 312527

70 or/63-69 1658778

71 animals/ not (humans/ and animals/) 5202533

72 Pregnancy/ or Hypertension, Pregnancy-Induced/ or Pregnancy Complications, Cardiovascular/ or exp Ocular Hypertension/ 1080947

73 (pregnancy or Pregnancy-Induced Hypertension or pregnancy Induced Hypertension or Gestational Hypertension or Pregnancy Transient Hypertension or maternal hypertension or cardiovascular pregnancy complication$ or Pregnancy Toxemia$ or Edema-Proteinuria-Hypertension Gestosis or Edema Proteinuria Hypertension Gestosis or Hypertension-Edema-Proteinuria Gestosis or Hypertension Edema Proteinuria Gestosis or Toxemia Of Pregnancy or Toxemia Of Pregnancies or EPH Complex or EPH Toxemia$ or EPH Gestosis or Proteinuria-Edema-Hypertension Gestosis or Proteinuria Edema Hypertension Gestosis or pre-eclampsia or pre eclampsia or preeclampsia or ocular hypertension$ or intraocular hypertension).ti. 224280

74 70 not (71 or 72 or 73) 1477139

75 exp cohort studies/ or exp epidemiologic studies/ or exp clinical trial/ or exp evaluation studies as topic/ or exp statistics as topic/ 6833675

76 ((control and (group* or study)) or (time and factors) or program or survey* or ci or cohort or comparative stud* or evaluation studies or follow-up*).mp. 9004942

77 or/75-76 11822983

78 (animals/ not humans/) or comment/ or editorial/ or exp review/ or meta analysis/ or consensus/ or exp guideline/ 9994723

79 hi.fs. or case report.mp. 745087

80 or/78-79 10643469

81 77 not 80 9268197

82 74 or 81 9724311

83 6 and 11 and 58 and 62 and 82 3858

----------------------------------------------------------------------

***************************

Database: Ovid Embase (1974 -- 01/07/2024)

Search date: 02/07/2024

Search Strategy:

----------------------------------------------------------------------

ID Search Hits

-----------------------------

1 exp aged/ 3878832

2 (advanced years or ageing or aging or elder$ or elderly or frail or geriatric? or gerontolog$ or later life or nursing care or nursing home? or old age or oldest old or pensioner? or post-menopausal or postmenopausal or senior or seniors or aged or septuagenarian$ or octogenarian$ or nonagenarian$ or centenarian$ or supercentenarian$ or senium).mp. 6671371

3 (old$ adj3 (adult? or female? or male? or men or people or person or women or patient?)).tw. 986166

4 ((over or old$) adj2 ("65" or "66" or "67" or "68" or "69" or "70" or "71" or "72" or "73" or "74" or "75" or "76" or "77" or "78" or "79" or "80" or "81" or "82" or "83" or "84" or "85" or "86" or "87" or "88" or "89" or "90" or "91" or "92" or "93" or "94" or "95" or "96" or "97" or "98" or "99" or "100") adj year?).tw. 58092

5 (aged or aging or ageing or elder$ or geriatric$ or gerontolog$).jx. 259718

6 or/1-5 7161114

7 exp hypertension/ 1034735

8 hypertens$.tw. 818452

9 ((elevat$ or high$ or raised) adj2 (bp or blood pressure)).tw. 75139

10 or/7-9 1306846

11 exp antihypertensive agent/ 961055

12 diuretic agent/ 96016

13 exp thiazide diuretic agent/ 61348

14 exp loop diuretic agent/ 87746

15 exp osmotic diuretic agent/ 98553

16 exp potassium sparing diuretic agent/ 61305

17 acetazolamide/ or ethoxzolamide/ or fenquizone/ or indapamide/ or tripamide/ 28183

18 (antihypertens$ or anti-hypertens$ or hypotensiv$).tw. 123194

19 ((ceiling or loop) adj diuretic?).tw. 6493

20 (amiloride or benzothiadiazine or bendroflumethiazide or bumetanide or chlorothiazide or cyclopenthiazide or furosemide or hydrochlorothiazide or hydroflumethiazide or methyclothiazide or metolazone or polythiazide or trichlormethiazide or veratide or thiazide?).tw. 51466

21 (chlorthalidone or chlortalidone or phthalamudine or chlorphthalidolone or oxodoline or thalitone or hygroton or indapamide or metindamide).tw. 4578

22 (Acetazolamide or azosemide or buthiazide or Canrenone or Chlorthalidone or cicletanine or Clopamide or Cyclopenthiazide or cyclothiazide or drospirenone or Eplerenone or Ethacrynic Acid or ethanolamine O-sulfate or Ethoxzolamide or ethylisopropylamiloride or etozolin or fenquizone or indacrinone or Indapamide or Isosorbide or Mannitol or Mefruside or Metolazone or "N-(3-(1-cyclopropyl-1-(2,4-difluorophenyl)ethyl)-1H-indol-7-yl)methanesulfonamide" or "N-4,4-dimethyl-2-thioxo-1,4-dihydro-2H-3,1-benzoxazin-6-ylthiophene-2-sulfonamide" or piretanide or quinethazone or Spironolactone or Torsemide or Triamterene or tripamide or Xipamide).tw. 61701

23 or/11-22 1166049

24 exp dipeptidyl carboxypeptidase inhibitor/ 211890

25 angiotensin converting enzyme inhibit$.tw. 30508

26 (ace adj2 inhibit$).tw. 34207

27 acei.tw. 10962

28 (alacepril or altiopril or ancovenin or benazepril or captopril or ceranapril or ceronapril or cilazapril or deacetylalacepril or delapril or derapril or enalapril or epicaptopril or fasidotril or fosinopril or foroxymithine or gemopatrilat or idapril or imidapril or indolapril or libenzapril or lisinopril or moexipril or moveltipril or omapatrilat or pentopril$ or perindopril$ or pivopril or quinapril$ or ramipril$ or rentiapril or saralasin or s nitrosocaptopril or spirapril$ or temocapril$ or teprotide or trandolapril$ or utibapril$ or zabicipril$ or zofenopril$ or Aceon or Accupril or Altace or Capoten or Lotensin or Mavik or Monopril or Prinivil or Univas or Vasotec or Zestril).tw. 43230

29 or/24-28 224573

30 exp angiotensin receptor antagonist/ 127952

31 (angiotensin adj3 receptor antagon$).tw. 5348

32 (angiotensin adj3 receptor block$).tw. 21504

33 (arb or arbs).tw. 19143

34 (abitesartan or azilsartan or candesartan or elisartan or embusartan or eprosartan or forasartan or irbesartan or losartan or milfasartan or olmesartan or saprisartan or tasosartan or telmisartan or valsartan or zolasartan or Atacand or Avapro or Benicar or Cozaar or Diovan or Micardis or Teveten).tw. 36332

35 or/30-34 138613

36 exp calcium channel blocking agent/ 351882

37 (amlodipine or aranidipine or barnidipine or bencyclane or benidipine or bepridil or cilnidipine or cinnarizine or clentiazem or darodipine or diltiazem or efonidipine or elgodipine or etafenone or fantofarone or felodipine or fendiline or flunarizine or gallopamil or isradipine or lacidipine or lercanidipine or lidoflazine or lomerizine or manidipine or mibefradil or nicardipine or nifedipine or niguldipine or nilvadipine or nimodipine or nisoldipine or nitrendipine or perhexiline or prenylamine or semotiadil or terodiline or tiapamil or verapamil or Cardizem CD or Dilacor XR or Tiazac or Cardizem Calan or Isoptin or Calan SR or Isoptin SR Coer or Covera HS or Verelan PM).tw. 90970

38 (calcium adj2 (antagonist? or block$ or inhibit$)).tw. 56191

39 or/36-38 374925

40 (methyldopa or alphamethyldopa or amodopa or dopamet or dopegyt or dopegit or dopegite or emdopa or hyperpax or hyperpaxa or methylpropionic acid or dopergit or meldopa or methyldopate or medopa or medomet or sembrina or aldomet or aldometil or aldomin or hydopa or methyldihydroxyphenylalanine or methyl dopa or mulfasin or presinol or presolisin or sedometil or sembrina or taquinil or dihydroxyphenylalanine or methylphenylalanine or methylalanine or alpha methyl dopa).mp. 29850

41 (reserpine or serpentina or rauwolfia or serpasil).mp. 23918

42 (clonidine or adesipress or arkamin or caprysin or catapres$ or catasan or chlofazolin or chlophazolin or clinidine or clofelin$ or clofenil or clomidine or clondine or clonistada or clonnirit or clophelin$ or dichlorophenylaminoimidazoline or dixarit or duraclon or gemiton or haemiton or hemiton or imidazoline or isoglaucon or klofelin or klofenil or m-5041t or normopresan or paracefan or st-155 or st 155 or tesno timelets).mp. 52231

43 (hydralazin$ or hydrallazin$ or hydralizine or hydrazinophtalazine or hydrazinophthalazine or hydrazinophtalizine or dralzine or hydralacin or hydrolazine or hypophthalin or hypoftalin or hydrazinophthalazine or idralazina or 1-hydrazinophthalazine or apressin or nepresol or apressoline or apresoline or apresolin or alphapress or alazine or idralazina or lopress or plethorit or praeparat).tw. 7182

44 or/40-43 102338

45 exp beta adrenergic receptor blocking agent/ 350268

46 (acebutolol or adimolol or afurolol or alprenolol or amosulalol or arotinolol or atenolol or befunolol or betaxolol or bevantolol or bisoprolol or bopindolol or bornaprolol or brefonalol or bucindolol or bucumolol or bufetolol or bufuralol or bunitrolol or bunolol or bupranolol or butofilolol or butoxamine or carazolol or carteolol or carvedilol or celiprolol or cetamolol or chlortalidone cloranolol or cyanoiodopindolol or cyanopindolol or deacetylmetipranolol or diacetolol or dihydroalprenolol or dilevalol or epanolol or esmolol or exaprolol or falintolol or flestolol or flusoxolol or hydroxybenzylpinodolol or hydroxycarteolol or hydroxymetoprolol or indenolol or iodocyanopindolol or iodopindolol or iprocrolol or isoxaprolol or labetalol or landiolol or levobunolol or levomoprolol or medroxalol or mepindolol or methylthiopropranolol or metipranolol or metoprolol or moprolol or nadolol or oxprenolol or penbutolol or pindolol or nadolol or nebivolol or nifenalol or nipradilol or oxprenolol or pafenolol or pamatolol or penbutolol or pindolol or practolol or primidolol or prizidilol or procinolol or pronetalol or propranolol or proxodolol or ridazolol or salcardolol or soquinolol or sotalol or spirendolol or talinolol or tertatolol or tienoxolol or tilisolol or timolol or tolamolol or toliprolol or tribendilol or xibenolol).tw. 94249

47 (beta adj2 (adrenergic? or antagonist? or block$ or receptor?)).tw. 145912

48 or/45-47 420184

49 exp alpha adrenergic receptor blocking agent/ 334981

50 (alfuzosin or bunazosin or doxazosin or metazosin or neldazosin or prazosin or silodosin or tamsulosin or terazosin or tiodazosin or trimazosin).tw. 19611

51 (adrenergic adj2 (alpha or antagonist?)).tw. 20199

52 ((adrenergic or alpha or receptor?) adj2 block$).tw. 89339

53 or/49-52 416462

54 (vasoactive antagonist$ or vasodilator$ or vasorelaxant$).tw. 54347

55 (ganglionic block$ or ganglioplegic agent$).tw. 1791

56 or/54-55 56049

57 23 or 29 or 35 or 39 or 44 or 48 or 53 or 56 1703727

58 drug withdrawal/ 270332

59 deprescription/ 2144

60 (deprescri$ or de-prescri$).tw. 3463

61 ((withhold$ or withheld or withdraw$ or discontinu$ or reduced or reduces or reducing or reduction? or ceased or ceasing or cessation? or stop$ or taper$) adj3 (antihypertens$ or anti-hypertens$ or diuretic? or dosage? or dose or doses or drug$ or medicat$ or prescri$ or treatment$ or therapy or therapies or therapeutics)).tw. 479603

62 or/58-61 698643

63 randomized controlled trial/ 829416

64 crossover procedure/ 78564

65 double-blind procedure/ 220679

66 (randomi?ed or randomly).tw. 1660679

67 (crossover$ or cross-over$).tw. 130937

68 placebo.ab. 369619

69 (doubl$ adj blind$).tw. 253436

70 assign$.ab. 509610

71 allocat$.ab. 211598

72 or/63-71 2373279

73 (exp animal/ or animal.hw. or nonhuman/) not (exp human/ or human cell/ or (human or humans).ti.) 7384947

74 exp pregnancy/ or maternal hypertension.mp. or exp pregnancy complication/ or intraocular hypertension/ 1144738

75 (pregnancy or Pregnancy-Induced Hypertension or pregnancy Induced Hypertension or Gestational Hypertension or Pregnancy Transient Hypertension or cardiovascular pregnancy complication$ or Pregnancy Toxemia$ or Edema-Proteinuria-Hypertension Gestosis or Edema Proteinuria Hypertension Gestosis or Hypertension-Edema-Proteinuria Gestosis or Hypertension Edema Proteinuria Gestosis or Toxemia Of Pregnancy or Toxemia Of Pregnancies or EPH Complex or EPH Toxemia$ or EPH Gestosis or Proteinuria-Edema-Hypertension Gestosis or Proteinuria Edema Hypertension Gestosis or pre-eclampsia or pre eclampsia or preeclampsia or ocular hypertension$ or intraocular hypertension).ti. 258994

76 hi.fs. or case report.mp. 3060628

77 Clinical article/ or controlled study/ or major clinical study/ or prospective study/ or cohort.mp. or compared.mp. or groups.mp. or multivariate.mp. 19083601

78 72 or 77 19579347

79 73 or 74 or 75 or 76 11325143

80 78 not 79 14346540

81 6 and 10 and 57 and 62 and 80 6003

----------------------------------------------------------------------

***************************

Database: Cochrane Library (1996 -- Present)

Search date: 02/07/2024

Search Strategy:

----------------------------------------------------------------------

ID Search Hits

-----------------------------

#1 MeSH descriptor: [Aged] explode all trees 276555

#2 (ageing OR aging OR elder* OR frail OR geriatric* OR nursing care OR nursing home* OR old age OR older OR pensioner* OR postmenopausal OR post‐menopausal OR senior* OR septuagenarian* OR octogenarian* OR nonagenarian* OR centenarian* OR supercentenarian* OR senium):ti,ab,kw 208868

#3 ((65 OR 66 OR 67 OR 68 OR 69 OR 70 OR 71 OR 72 OR 73 OR 74 OR 75 OR 76 OR 77 OR 78 OR 79 OR 80 OR 81 OR 82 OR 83 OR 84 OR 85 OR 86 OR 87 OR 88 OR 89 OR 90 OR 91 OR 92 OR 93 OR 94 OR 95 OR 96 OR 97 OR 98 OR 99 OR 100) NEXT year?):ti,ab,kw 140820

#4 (old* NEAR/3 (adult? or female? or male? or men or people or person? or women or patient?)):ti,ab,kw 57955

#5 (aged OR aging OR ageing OR elder or geriatric* or gerontolog*):so 15322

#6 {OR #1-#5} 511392

#7 MeSH descriptor: [Hypertension] this term only 23661

#8 MeSH descriptor: [Essential Hypertension] this term only 400

#9 (hypertens* OR elevated blood pressure OR high* blood pressure OR raised blood pressure):ti,ab,kw 117929

#10 {OR #7-#9} 117929

#11 MeSH descriptor: [Antihypertensive Agents] explode all trees 10863

#12 MeSH descriptor: [Diuretics] explode all trees 4122

#13 MeSH descriptor: [Thiazides] explode all trees 3070

#14 (antihypertensiv* OR anti-hypertensiv* OR hypotensiv* OR thiazide* OR sodium chloride symporter inhibit* OR sodium potassium chloride symporter inhibit* OR ceiling diuretic* OR loop diuretic*):ti,ab,kw 28012

#15 (amiloride OR benzothiadiazine OR bendroflumethiazide OR bumetanide OR chlorothiazide OR cyclopenthiazide OR furosemide OR hydrochlorothiazide OR hydroflumethiazide OR methyclothiazide OR metolazone OR polythiazide OR trichlormethiazide OR veratide OR thiazide*):ti,ab,kw 8805

#16 (chlorthalidone OR chlortalidone OR phthalamudine OR chlorphthalidolone OR oxodoline OR thalitone OR hygroton OR indapamide OR metindamide):ti,ab,kw 1620

#17 (Acetazolamide or azosemide or buthiazide or Canrenone or Chlorthalidone or cicletanine or Clopamide or Cyclopenthiazide or cyclothiazide or drospirenone or Eplerenone or Ethacrynic Acid or ethanolamine O-sulfate or Ethoxzolamide or ethylisopropylamiloride or etozolin or fenquizone or indacrinone or Indapamide or Isosorbide or Mannitol or Mefruside or Metolazone or "N-(3-(1-cyclopropyl-1-(2,4-difluorophenyl)ethyl)-1H-indol-7-yl)methanesulfonamide" or "N-4,4-dimethyl-2-thioxo-1,4-dihydro-2H-3,1-benzoxazin-6-ylthiophene-2-sulfonamide" or piretanide or quinethazone or Spironolactone or Torsemide or Triamterene or tripamide or Xipamide):ti,ab,kw 10711

#18 MeSH descriptor: [Angiotensin-Converting Enzyme Inhibitors] explode all trees 5306

#19 (angiotensin-converting enzyme inhibit* OR angiotensin converting enzyme inhibit* OR acei):ti,ab,kw 9537

#20 (ace NEAR/2 inhibit*):ti,ab,kw 4753

#21 (alacepril OR altiopril OR ancovenin OR benazepril OR captopril OR ceranapril OR ceronapril OR cilazapril OR deacetylalacepril OR delapril OR derapril OR enalapril OR epicaptopril OR fasidotril OR fosinopril OR foroxymithine OR gemopatrilat OR idapril OR imidapril OR indolapril OR libenzapril OR lisinopril OR moexipril OR moveltipril OR omapatrilat OR pentopril* OR perindopril* OR pivopril OR quinapril* OR ramipril* OR rentiapril OR saralasin OR s nitrosocaptopril OR spirapril* OR temocapril* OR teprotide OR trandolapril* OR utibapril* OR zabicipril* OR zofenopril* OR Aceon OR Accupril OR Altace OR Capoten OR Lotensin OR Mavik OR Monopril OR Prinivil OR Univas OR Vasotec OR Zestril):ti,ab,kw 10858

#22 MeSH descriptor: [Angiotensin Receptor Antagonists] explode all trees 3035

#23 (Angiotensin Receptor Antagonist* OR Angiotensin Receptor block* OR arb OR arbs):ti,ab,kw 8567

#24 (abitesartan OR azilsartan OR candesartan OR elisartan OR embusartan OR eprosartan OR forasartan OR irbesartan OR losartan OR milfasartan OR olmesartan OR saprisartan OR tasosartan OR telmisartan OR valsartan OR zolasartan OR Atacand OR Avapro OR Benicar OR Cozaar OR Diovan OR Micardis OR Teveten):ti,ab,kw 9677

#25 MeSH descriptor: [Calcium Channel Blockers] explode all trees 3529

#26 (calcium NEAR/2 (antagonist* OR block* OR inhibit*)):ti,ab,kw 8372

#27 (amlodipine OR aranidipine OR barnidipine OR bencyclane OR benidipine OR bepridil OR cilnidipine OR cinnarizine OR clentiazem OR darodipine OR diltiazem OR efonidipine OR elgodipine OR etafenone OR fantofarone OR felodipine OR fendiline OR flunarizine OR gallopamil OR isradipine OR lacidipine OR lercanidipine OR lidoflazine OR lomerizine OR manidipine OR mibefradil OR nicardipine OR nifedipine OR niguldipine OR nilvadipine OR nimodipine OR nisoldipine OR nitrendipine OR perhexiline OR prenylamine OR semotiadil OR terodiline OR tiapamil OR verapamil OR Cardizem CD OR Dilacor XR OR Tiazac OR Cardizem Calan OR Isoptin OR Calan SR OR Isoptin SR Coer OR Covera HS OR Verelan PM):ti,ab,kw 16372

#28 (methyldopa OR alphamethyldopa OR amodopa OR dopamet OR dopegyt OR dopegit OR dopegite OR emdopa OR hyperpax OR hyperpaxa OR methylpropionic acid OR dopergit OR meldopa OR methyldopate OR medopa OR medomet OR sembrina OR aldomet OR aldometil OR aldomin OR hydopa OR methyldihydroxyphenylalanine OR methyl dopa OR mulfasin OR presinol OR presolisin OR sedometil OR sembrina OR taquinil OR dihydroxyphenylalanine OR methylphenylalanine OR methylalanine OR alpha methyl dopa):ti,ab,kw 993

#29 (reserpine OR serpentina OR rauwolfia OR serpasil):ti,ab,kw 351

#30 (clonidine OR adesipress OR arkamin OR caprysin OR catapres* OR catasan OR chlofazolin OR chlophazolin OR clinidine OR clofelin* OR clofenil OR clomidine OR clondine OR clonistada OR clonnirit OR clophelin* OR dichlorophenylaminoimidazoline OR dixarit OR duraclon OR gemiton OR haemiton OR hemiton OR imidazoline OR isoglaucon OR klofelin OR klofenil OR m-5041t OR normopresan OR paracefan OR st-155 OR st 155 OR tesno timelets):ti,ab,kw 5168

#31 MeSH descriptor: [Hydralazine] explode all trees 407

#32 (hydralazin* OR hydrallazin* OR hydralizine OR hydrazinophtalazine OR hydrazinophthalazine OR hydrazinophtalizine OR dralzine OR hydralacin* OR hydrolazine OR hypophthalin OR hypoftalin OR hydrazinophthalazine OR idralazina OR *hydrazinophthalazine OR apressin OR nepresol OR apressoline OR apresoline OR apresolin OR alphapress OR alazine OR idralazina OR lopress OR plethorit OR praeparat):ti,ab,kw 705

#33 MeSH descriptor: [Adrenergic beta-Antagonists] explode all trees 5812

#34 (beta NEAR/2 (adrenergic* OR antagonist* OR block* OR receptor*)):ti,ab,kw 18484

#35 (adrenergic NEAR/2 (beta OR antagonist*)):ti,ab,kw 13358

#36 ((adrenergic* or beta or receptor*) NEAR/2 block*):ti,ab,kw 19461

#37 (acebutolol OR adimolol OR afurolol OR alprenolol OR amosulalol OR arotinolol OR atenolol OR befunolol OR betaxolol OR bevantolol OR bisoprolol OR bopindolol OR bornaprolol OR brefonalol OR bucindolol OR bucumolol OR bufetolol OR bufuralol OR bunitrolol OR bunolol OR bupranolol OR butofilolol OR butoxamine OR carazolol OR carteolol OR carvedilol OR celiprolol OR cetamolol OR chlortalidone cloranolol OR cyanoiodopindolol OR cyanopindolol OR deacetylmetipranolol OR diacetolol OR dihydroalprenolol OR dilevalol OR epanolol OR esmolol OR exaprolol OR falintolol OR flestolol OR flusoxolol OR hydroxybenzylpinodolol OR hydroxycarteolol OR hydroxymetoprolol OR indenolol OR iodocyanopindolol OR iodopindolol OR iprocrolol OR isoxaprolol OR labetalol OR landiolol OR levobunolol OR levomoprolol OR medroxalol OR mepindolol OR methylthiopropranolol OR metipranolol OR metoprolol OR moprolol OR nadolol OR oxprenolol OR penbutolol OR pindolol OR nadolol OR nebivolol OR nifenalol OR nipradilol OR oxprenolol OR pafenolol OR pamatolol OR penbutolol OR pindolol OR practolol OR primidolol OR prizidilol OR procinolol OR pronetalol OR propranolol OR proxodolol OR ridazolol OR salcardolol OR soquinolol OR sotalol OR spirendolol OR talinolol OR tertatolol OR tienoxolol OR tilisolol OR timolol OR tolamolol OR toliprolol OR tribendilol OR xibenolol):ti,ab,kw 19909

#38 MeSH descriptor: [Adrenergic alpha-Antagonists] explode all trees 1560

#39 (alpha NEAR/2 (adrenergic* OR antagonist* OR block* OR receptor*)):ti,ab,kw 7156

#40 (adrenergic NEAR/2 (alpha OR antagonist*)):ti,ab,kw 9676

#41 ((adrenergic* or alpha or receptor*) NEAR/2 block*):ti,ab,kw 13119

#42 (alfuzosin OR bunazosin OR doxazosin OR metazosin OR neldazosin OR prazosin OR silodosin OR tamsulosin OR terazosin OR tiodazosin OR trimazosin):ti,ab,kw 3755

#43 MeSH descriptor: [Vasodilator Agents] this term only 4604

#44 (vasoactive antagonist* or vasodilator* or vasorelaxant*):ti,ab,kw 8435

#45 MeSH descriptor: [Ganglionic Blockers] this term only 21

#46 (ganglionic block* or ganglioplegic agent*):ti,ab,kw 79

#47 {OR #11-#46} 104058

#48 MeSH descriptor: [Deprescriptions] this term only 110

#49 MeSH descriptor: [Drug Prescriptions] this term only 754

#50 MeSH descriptor: [Withholding Treatment] this term only 562

#51 (deprescri* or de-prescri*):ti,ab,kw 405

#52 ((withhold* or withheld or withdraw* or discontinu* or reduced or reduces or reducing or reduction? or ceased or ceasing or cessation? or stop* or taper*) NEAR/3 (antihypertens* or anti-hypertens* or hypotensiv* or diuretic? or dosage? or dose or doses or drug* or medicat* or prescri* or treatment* or therapy or therapies or therapeutics)):ti,ab,kw 103545

#53 {OR #48-#52} 104356

#54 {AND #6, #10, #47, #53} 2349

#55 MeSH descriptor: [Animals] explode all trees 896092

#56 MeSH descriptor: [Humans] explode all trees 892428

#57 #55 NOT #56 3664

#58 MeSH descriptor: [Pregnancy] this term only 33582

#59 MeSH descriptor: [Hypertension, Pregnancy-Induced] this term only 385

#60 MeSH descriptor: [Pregnancy Complications, Cardiovascular] this term only 412

#61 MeSH descriptor: [Ocular Hypertension] explode all trees 4598

#62 (pregnancy OR Pregnancy-Induced Hypertension OR pregnancy Induced Hypertension OR Gestational Hypertension OR Pregnancy Transient Hypertension OR maternal hypertension OR cardiovascular pregnancy complication$ OR Pregnancy Toxemia$ OR Edema-Proteinuria-Hypertension Gestosis OR Edema Proteinuria Hypertension Gestosis OR Hypertension-Edema-Proteinuria Gestosis OR Hypertension Edema Proteinuria Gestosis OR Toxemia Of Pregnancy OR Toxemia Of Pregnancies OR EPH Complex OR EPH Toxemia$ OR EPH Gestosis OR Proteinuria-Edema-Hypertension Gestosis OR Proteinuria Edema Hypertension Gestosis OR pre-eclampsia OR pre eclampsia OR preeclampsia OR ocular hypertension$ OR intraocular hypertension):ti,kw 56883

#63 {OR #57-#62} 63229

#64 #54 NOT #63 2223

----------------------------------------------------------------------

***************************

Database: CINAHL with Full Text (1963 -- Present)

Expanders - Apply equivalent subjects

Search modes - Find all my search terms

Interface - EBSCOhost Research Databases

Search Screen - Advanced Search

Search date: 02/07/2024

Search Strategy:

----------------------------------------------------------------------

S1 (MH "Aged+") 961020

S2 (TI "advanced years") OR (TI "ageing") OR (TI "aging") OR (TI "elder*") OR (TI "frail") OR (TI "geriatric*") OR (TI "gerontolog*") OR (TI "later life") OR (TI "nursing care") OR (TI "nursing home*") OR (TI "old age") OR (TI "oldest old") OR (TI "pensioner*") OR (TI "post-menopausal") OR (TI "postmenopausal") OR (TI "senior*") OR (TI "aged") OR (TI "septuagenarian#") OR (TI "octogenarian#") OR (TI "nonagenarian#") OR (TI "centenarian#") OR (TI "supercentenarian#") OR (TI "senium") OR (TI old* N2 ("adult#" OR "female#" OR "male#" OR "men" OR "people" OR "person" OR "women" OR "patient#")) OR (AB "advanced years") OR (AB "ageing") OR (AB "aging") OR (AB "elder*") OR (AB "frail") OR (AB "geriatric*") OR (AB "gerontolog*") OR (AB "later life") OR (AB "nursing care") OR (AB "nursing home*") OR (AB "old age") OR (AB "oldest old") OR (AB "pensioner*") OR (AB "post-menopausal") OR (AB "postmenopausal") OR (AB "senior*") OR (AB "aged") OR (AB "septuagenarian#") OR (AB "octogenarian#") OR (AB "nonagenarian#") OR (AB "centenarian#") OR (AB "supercentenarian#") OR (AB "senium") OR (AB old* N2 ("adult#" OR "female#" OR "male#" OR "men" OR "people" OR "person" OR "women" OR "patient#")) OR (AG "Aged") OR (AG "Aged, 80 and Over") OR (AG "Aged, Hospitalized") OR (AG "Frail Elderly") OR (SO aged) OR (SO aging) OR (SO ageing) OR (SO elder*) OR (SO geriatric*) OR (SO gerontolog*) 1791784

S3 (MH "Hypertension") OR (MH "Essential Hypertension") 66733

S4 (TI "hypertens*") OR (TI (("elevat*" OR "high*" OR "raised") N1 ("bp" OR "blood pressure"))) OR (AB "hypertens*") OR (AB (("elevat*" OR "high*" OR "raised") N1 ("bp" OR "blood pressure"))) 120691

S5 (MH "Antihypertensive Agents+") OR (MH "Diuretics+") OR (MH "Vasodilator Agents") OR (MH "Ganglionic Blockers") 47461

S6 (TI "antihypertens*") OR (TI "anti-hypertens*") OR (TI "hypotensiv*") OR "(TI ("ceiling" OR "loop") N1 diuretic#) OR (TI "vasoactive antagonist*") OR (TI vasodilator*) OR (TI vasorelaxant*) OR (TI "ganglionic block*") OR (TI "ganglioplegic agent*") OR (TI amiloride) OR (TI benzothiadiazine) OR (TI bendroflumethiazide) OR (TI bumetanide) OR (TI chlorothiazide) OR (TI cyclopenthiazide) OR (TI furosemide) OR (TI hydrochlorothiazide) OR (TI hydroflumethiazide) OR (TI methyclothiazide) OR (TI metolazone) OR (TI polythiazide) OR (TI trichlormethiazide) OR (TI veratide) OR (TI thiazide#) OR (TI chlorthalidone) OR (TI chlortalidone) OR (TI phthalamudine) OR (TI chlorphthalidolone) OR (TI oxodoline) OR (TI thalitone) OR (TI hygroton) OR (TI indapamide) OR (TI metindamide) OR (AB "antihypertens*") OR (AB "anti-hypertens*") OR (AB "hypotensiv*") OR (AB "vasoactive antagonist*") OR (AB vasodilator*) OR (AB vasorelaxant*) OR (AB "ganglionic block*") OR (AB "ganglioplegic agent*") OR (AB ("ceiling" OR "loop") N1 diuretic#) OR (AB amiloride) OR (AB benzothiadiazine) OR (AB bendroflumethiazide) OR (AB bumetanide) OR (AB chlorothiazide) OR (AB cyclopenthiazide) OR (AB furosemide) OR (AB hydrochlorothiazide) OR (AB hydroflumethiazide) OR (AB methyclothiazide) OR (AB metolazone) OR (AB polythiazide) OR (AB trichlormethiazide) OR (AB veratide) OR (AB thiazide#) OR (AB chlorthalidone) OR (AB chlortalidone) OR (AB phthalamudine) OR (AB chlorphthalidolone) OR (AB oxodoline) OR (AB thalitone) OR (AB hygroton) OR (AB indapamide) OR (AB metindamide) 7010

S7 (MH "Angiotensin-Converting Enzyme Inhibitors+") 10929

S8 (TI ("angiotensin converting enzyme" N1 inhibit*)) OR (TI (ACE N1 inhibit*)) OR (TI ACEI) OR (TI alacepril) OR (TI altiopril) OR (TI ancovenin) OR (TI benazepril) OR (TI captopril) OR (TI ceranapril) OR (TI ceronapril) OR (TI cilazapril) OR (TI deacetylalacepril) OR (TI delapril) OR (TI derapril) OR (TI enalapril) OR (TI epicaptopril) OR (TI fasidotril) OR (TI fosinopril) OR (TI foroxymithine) OR (TI gemopatrilat) OR (TI idapril) OR (TI imidapril) OR (TI indolapril) OR (TI libenzapril) OR (TI lisinopril) OR (TI moexipril) OR (TI moveltipril) OR (TI omapatrilat) OR (TI pentopril*) OR (TI perindopril*) OR (TI pivopril) OR (TI quinapril*) OR (TI ramipril*) OR (TI rentiapril) OR (TI saralasin) OR (TI s nitrosocaptopril) OR (TI spirapril*) OR (TI temocapril*) OR (TI teprotide) OR (TI trandolapril*) OR (TI utibapril*) OR (TI zabicipril*) OR (TI zofenopril*) OR (TI Aceon) OR (TI Accupril) OR (TI Altace) OR (TI Capoten) OR (TI Lotensin) OR (TI Mavik) OR (TI Monopril) OR (TI Prinivil) OR (TI Univas) OR (TI Vasotec) OR (TI Zestril) OR (AB ("angiotensin converting enzyme" N1 inhibit*)) OR (AB (ACE N1 inhibit*)) OR (AB ACEI) OR (AB alacepril) OR (AB altiopril) OR (AB ancovenin) OR (AB benazepril) OR (AB captopril) OR (AB ceranapril) OR (AB ceronapril) OR (AB cilazapril) OR (AB deacetylalacepril) OR (AB delapril) OR (AB derapril) OR (AB enalapril) OR (AB epicaptopril) OR (AB fasidotril) OR (AB fosinopril) OR (AB foroxymithine) OR (AB gemopatrilat) OR (AB idapril) OR (AB imidapril) OR (AB indolapril) OR (AB libenzapril) OR (AB lisinopril) OR (AB moexipril) OR (AB moveltipril) OR (AB omapatrilat) OR (AB pentopril*) OR (AB perindopril*) OR (AB pivopril) OR (AB quinapril*) OR (AB ramipril*) OR (AB rentiapril) OR (AB saralasin) OR (AB s nitrosocaptopril) OR (AB spirapril*) OR (AB temocapril*) OR (AB teprotide) OR (AB trandolapril*) OR (AB utibapril*) OR (AB zabicipril*) OR (AB zofenopril*) OR (AB Aceon) OR (AB Accupril) OR (AB Altace) OR (AB Capoten) OR (AB Lotensin) OR (AB Mavik) OR (AB Monopril) OR (AB Prinivil) OR (AB Univas) OR (AB Vasotec) OR (AB Zestril) 10344

S9 (MH "Angiotensin II Type I Receptor Blockers+") 6159

S10 (TI (angiotensin N2 "receptor antagon*")) OR (TI (angiotensin N2 "receptor block*") OR (TI arb) or (TI arbs) OR (TI abitesartan) OR (TI azilsartan) OR (TI candesartan) OR (TI elisartan) OR (TI embusartan) OR (TI eprosartan) OR (TI forasartan) OR (TI irbesartan) OR (TI losartan) OR (TI milfasartan) OR (TI olmesartan) OR (TI saprisartan) OR (TI tasosartan) OR (TI telmisartan) OR (TI valsartan) OR (TI zolasartan) OR (TI Atacand) OR (TI Avapro) OR (TI Benicar) OR (TI Cozaar) OR (TI Diovan) OR (TI Micardis) OR (TI Teveten) OR (AB (angiotensin N2 "receptor antagon*")) OR (AB (angiotensin N2 "receptor block*") OR (AB arb) or (AB arbs) OR (AB abitesartan) OR (AB azilsartan) OR (AB candesartan) OR (AB elisartan) OR (AB embusartan) OR (AB eprosartan) OR (AB forasartan) OR (AB irbesartan) OR (AB losartan) OR (AB milfasartan) OR (AB olmesartan) OR (AB saprisartan) OR (AB tasosartan) OR (AB telmisartan) OR (AB valsartan) OR (AB zolasartan) OR (AB Atacand) OR (AB Avapro) OR (AB Benicar) OR (AB Cozaar) OR (AB Diovan) OR (AB Micardis) OR (AB Teveten) 8348

S11 (MH "Calcium Channel Blockers+") 7804

S12 (TI amlodipine) OR (TI aranidipine) OR (TI barnidipine) OR (TI bencyclane) OR (TI benidipine) OR (TI bepridil) OR (TI cilnidipine) OR (TI cinnarizine) OR (TI clentiazem) OR (TI darodipine) OR (TI diltiazem) OR (TI efonidipine) OR (TI elgodipine) OR (TI etafenone) OR (TI fantofarone) OR (TI felodipine) OR (TI fendiline) OR (TI flunarizine) OR (TI gallopamil) OR (TI isradipine) OR (TI lacidipine) OR (TI lercanidipine) OR (TI lidoflazine) OR (TI lomerizine) OR (TI manidipine) OR (TI mibefradil) OR (TI nicardipine) OR (TI nifedipine) OR (TI niguldipine) OR (TI nilvadipine) OR (TI nimodipine) OR (TI nisoldipine) OR (TI nitrendipine) OR (TI perhexiline) OR (TI prenylamine) OR (TI semotiadil) OR (TI terodiline) OR (TI tiapamil) OR (TI verapamil) OR (TI Cardizem CD) OR (TI Dilac OR (TI XR) OR (TI Tiazac) OR (TI Cardizem Calan) OR (TI Isoptin) OR (TI Calan SR) OR (TI Isoptin SR Coer) OR (TI Covera HS) OR (TI Verelan PM) OR (TI (calcium N1 (antagonist* OR block* OR inhibit*))) OR (AB amlodipine) OR (AB aranidipine) OR (AB barnidipine) OR (AB bencyclane) OR (AB benidipine) OR (AB bepridil) OR (AB cilnidipine) OR (AB cinnarizine) OR (AB clentiazem) OR (AB darodipine) OR (AB diltiazem) OR (AB efonidipine) OR (AB elgodipine) OR (AB etafenone) OR (AB fantofarone) OR (AB felodipine) OR (AB fendiline) OR (AB flunarizine) OR (AB gallopamil) OR (AB isradipine) OR (AB lacidipine) OR (AB lercanidipine) OR (AB lidoflazine) OR (AB lomerizine) OR (AB manidipine) OR (AB mibefradil) OR (AB nicardipine) OR (AB nifedipine) OR (AB niguldipine) OR (AB nilvadipine) OR (AB nimodipine) OR (AB nisoldipine) OR (AB nitrendipine) OR (AB perhexiline) OR (AB prenylamine) OR (AB semotiadil) OR (AB terodiline) OR (AB tiapamil) OR (AB verapamil) OR (AB Cardizem CD) OR (AB Dilac OR (AB XR) OR (AB Tiazac) OR (AB Cardizem Calan) OR (AB Isoptin) OR (AB Calan SR) OR (AB Isoptin SR Coer) OR (AB Covera HS) OR (AB Verelan PM) OR (AB (calcium N1 (antagonist* OR block* OR inhibit*))) 9672

S13 (TI methyldopa) OR (TI alphamethyldopa) OR (TI amodopa) OR (TI dopamet) OR (TI dopegyt) OR (TI dopegit) OR (TI dopegite) OR (TI emdopa) OR (TI hyperpax) OR (TI hyperpaxa) OR (TI "methylpropionic acid") OR (TI dopergit) OR (TI meldopa) OR (TI methyldopate) OR (TI medopa) OR (TI medomet) OR (TI sembrina) OR (TI aldomet) OR (TI aldometil) OR (TI aldomin) OR (TI hydopa) OR (TI methyldihydroxyphenylalanine) OR (TI methyl dopa) OR (TI mulfasin) OR (TI presinol) OR (TI presolisin) OR (TI sedometil) OR (TI sembrina) OR (TI taquinil) OR (TI dihydroxyphenylalanine) OR (TI methylphenylalanine) OR (TI methylalanine) OR (TI alpha methyl dopa) OR (TI reserpine) OR (TI serpentina) OR (TI rauwolfia) OR (TI serpasil) OR (TI clonidine) OR (TI adesipress) OR (TI arkamin) OR (TI caprysin) OR (TI catapres$) OR (TI catasan) OR (TI chlofazolin) OR (TI chlophazolin) OR (TI clinidine) OR (TI clofelin$) OR (TI clofenil) OR (TI clomidine) OR (TI clondine) OR (TI clonistada) OR (TI clonnirit) OR (TI clophelin$) OR (TI dichlorophenylaminoimidazoline) OR (TI dixarit) OR (TI duraclon) OR (TI gemiton) OR (TI haemiton) OR (TI hemiton) OR (TI imidazoline) OR (TI isoglaucon) OR (TI klofelin) OR (TI klofenil) OR (TI m-5041t) OR (TI normopresan) OR (TI paracefan) OR (TI st-155) OR (TI st 155) OR (TI tesno timelets) OR (AB methyldopa) OR (AB alphamethyldopa) OR (AB amodopa) OR (AB dopamet) OR (AB dopegyt) OR (AB dopegit) OR (AB dopegite) OR (AB emdopa) OR (AB hyperpax) OR (AB hyperpaxa) OR (AB "methylpropionic acid") OR (AB dopergit) OR (AB meldopa) OR (AB methyldopate) OR (AB medopa) OR (AB medomet) OR (AB sembrina) OR (AB aldomet) OR (AB aldometil) OR (AB aldomin) OR (AB hydopa) OR (AB methyldihydroxyphenylalanine) OR (AB methyl dopa) OR (AB mulfasin) OR (AB presinol) OR (AB presolisin) OR (AB sedometil) OR (AB sembrina) OR (AB taquinil) OR (AB dihydroxyphenylalanine) OR (AB methylphenylalanine) OR (AB methylalanine) OR (AB alpha methyl dopa) OR (AB reserpine) OR (AB serpentina) OR (AB rauwolfia) OR (AB serpasil) OR (AB clonidine) OR (AB adesipress) OR (AB arkamin) OR (AB caprysin) OR (AB catapres$) OR (AB catasan) OR (AB chlofazolin) OR (AB chlophazolin) OR (AB clinidine) OR (AB clofelin$) OR (AB clofenil) OR (AB clomidine) OR (AB clondine) OR (AB clonistada) OR (AB clonnirit) OR (AB clophelin$) OR (AB dichlorophenylaminoimidazoline) OR (AB dixarit) OR (AB duraclon) OR (AB gemiton) OR (AB haemiton) OR (AB hemiton) OR (AB imidazoline) OR (AB isoglaucon) OR (AB klofelin) OR (AB klofenil) OR (AB m-5041t) OR (AB normopresan) OR (AB paracefan) OR (AB st-155) OR (AB st 155) OR (AB tesno timelets) OR (SU methyldopa) OR (SU alphamethyldopa) OR (SU amodopa) OR (SU dopamet) OR (SU dopegyt) OR (SU dopegit) OR (SU dopegite) OR (SU emdopa) OR (SU hyperpax) OR (SU hyperpaxa) OR (SU "methylpropionic acid") OR (SU dopergit) OR (SU meldopa) OR (SU methyldopate) OR (SU medopa) OR (SU medomet) OR (SU sembrina) OR (SU aldomet) OR (SU aldometil) OR (SU aldomin) OR (SU hydopa) OR (SU methyldihydroxyphenylalanine) OR (SU methyl dopa) OR (SU mulfasin) OR (SU presinol) OR (SU presolisin) OR (SU sedometil) OR (SU sembrina) OR (SU taquinil) OR (SU dihydroxyphenylalanine) OR (SU methylphenylalanine) OR (SU methylalanine) OR (SU alpha methyl dopa) OR (SU reserpine) OR (SU serpentina) OR (SU rauwolfia) OR (SU serpasil) OR (SU clonidine) OR (SU adesipress) OR (SU arkamin) OR (SU caprysin) OR (SU catapres$) OR (SU catasan) OR (SU chlofazolin) OR (SU chlophazolin) OR (SU clinidine) OR (SU clofelin$) OR (SU clofenil) OR (SU clomidine) OR (SU clondine) OR (SU clonistada) OR (SU clonnirit) OR (SU clophelin$) OR (SU dichlorophenylaminoimidazoline) OR (SU dixarit) OR (SU duraclon) OR (SU gemiton) OR (SU haemiton) OR (SU hemiton) OR (SU imidazoline) OR (SU isoglaucon) OR (SU klofelin) OR (SU klofenil) OR (SU m-5041t) OR (SU normopresan) OR (SU paracefan) OR (SU st-155) OR (SU st 155) OR (SU tesno timelets) OR (MW methyldopa) OR (MW alphamethyldopa) OR (MW amodopa) OR (MW dopamet) OR (MW dopegyt) OR (MW dopegit) OR (MW dopegite) OR (MW emdopa) OR (MW hyperpax) OR (MW hyperpaxa) OR (MW "methylpropionic acid") OR (MW dopergit) OR (MW meldopa) OR (MW methyldopate) OR (MW medopa) OR (MW medomet) OR (MW sembrina) OR (MW aldomet) OR (MW aldometil) OR (MW aldomin) OR (MW hydopa) OR (MW methyldihydroxyphenylalanine) OR (MW methyl dopa) OR (MW mulfasin) OR (MW presinol) OR (MW presolisin) OR (MW sedometil) OR (MW sembrina) OR (MW taquinil) OR (MW dihydroxyphenylalanine) OR (MW methylphenylalanine) OR (MW methylalanine) OR (MW alpha methyl dopa) OR (MW reserpine) OR (MW serpentina) OR (MW rauwolfia) OR (MW serpasil) OR (MW clonidine) OR (MW adesipress) OR (MW arkamin) OR (MW caprysin) OR (MW catapres$) OR (MW catasan) OR (MW chlofazolin) OR (MW chlophazolin) OR (MW clinidine) OR (MW clofelin$) OR (MW clofenil) OR (MW clomidine) OR (MW clondine) OR (MW clonistada) OR (MW clonnirit) OR (MW clophelin$) OR (MW dichlorophenylaminoimidazoline) OR (MW dixarit) OR (MW duraclon) OR (MW gemiton) OR (MW haemiton) OR (MW hemiton) OR (MW imidazoline) OR (MW isoglaucon) OR (MW klofelin) OR (MW klofenil) OR (MW m-5041t) OR (MW normopresan) OR (MW paracefan) OR (MW st-155) OR (MW st 155) OR (MW tesno timelets) OR (OS methyldopa) OR (OS alphamethyldopa) OR (OS amodopa) OR (OS dopamet) OR (OS dopegyt) OR (OS dopegit) OR (OS dopegite) OR (OS emdopa) OR (OS hyperpax) OR (OS hyperpaxa) OR (OS "methylpropionic acid") OR (OS dopergit) OR (OS meldopa) OR (OS methyldopate) OR (OS medopa) OR (OS medomet) OR (OS sembrina) OR (OS aldomet) OR (OS aldometil) OR (OS aldomin) OR (OS hydopa) OR (OS methyldihydroxyphenylalanine) OR (OS methyl dopa) OR (OS mulfasin) OR (OS presinol) OR (OS presolisin) OR (OS sedometil) OR (OS sembrina) OR (OS taquinil) OR (OS dihydroxyphenylalanine) OR (OS methylphenylalanine) OR (OS methylalanine) OR (OS alpha methyl dopa) (OS reserpine) OR (OS serpentina) OR (OS rauwolfia) OR (OS serpasil) OR (OS clonidine) OR (OS adesipress) OR (OS arkamin) OR (OS caprysin) OR (OS catapres$) OR (OS catasan) OR (OS chlofazolin) OR (OS chlophazolin) OR (OS clinidine) OR (OS clofelin$) OR (OS clofenil) OR (OS clomidine) OR (OS clondine) OR (OS clonistada) OR (OS clonnirit) OR (OS clophelin$) OR (OS dichlorophenylaminoimidazoline) OR (OS dixarit) OR (OS duraclon) OR (OS gemiton) OR (OS haemiton) OR (OS hemiton) OR (OS imidazoline) OR (OS isoglaucon) OR (OS klofelin) OR (OS klofenil) OR (OS m-5041t) OR (OS normopresan) OR (OS paracefan) OR (OS st-155) OR (OS st 155) OR (OS tesno timelets) OR (DS methyldopa) OR (DS alphamethyldopa) OR (DS amodopa) OR (DS dopamet) OR (DS dopegyt) OR (DS dopegit) OR (DS dopegite) OR (DS emdopa) OR (DS hyperpax) OR (DS hyperpaxa) OR (DS "methylpropionic acid") OR (DS dopergit) OR (DS meldopa) OR (DS methyldopate) OR (DS medopa) OR (DS medomet) OR (DS sembrina) OR (DS aldomet) OR (DS aldometil) OR (DS aldomin) OR (DS hydopa) OR (DS methyldihydroxyphenylalanine) OR (DS methyl dopa) OR (DS mulfasin) OR (DS presinol) OR (DS presolisin) OR (DS sedometil) OR (DS sembrina) OR (DS taquinil) OR (DS dihydroxyphenylalanine) OR (DS methylphenylalanine) OR (DS methylalanine) OR (DS alpha methyl dopa) OR (DS reserpine) OR (DS serpentina) OR (DS rauwolfia) OR (DS serpasil) OR (DS clonidine) OR (DS adesipress) OR (DS arkamin) OR (DS caprysin) OR (DS catapres$) OR (DS catasan) OR (DS chlofazolin) OR (DS chlophazolin) OR (DS clinidine) OR (DS clofelin$) OR (DS clofenil) OR (DS clomidine) OR (DS clondine) OR (DS clonistada) OR (DS clonnirit) OR (DS clophelin$) OR (DS dichlorophenylaminoimidazoline) OR (DS dixarit) OR (DS duraclon) OR (DS gemiton) OR (DS haemiton) OR (DS hemiton) OR (DS imidazoline) OR (DS isoglaucon) OR (DS klofelin) OR (DS klofenil) OR (DS m-5041t) OR (DS normopresan) OR (DS paracefan) OR (DS st-155) OR (DS st 155) OR (DS tesno timelets) 2993

S14 (MH "Hydralazine+") 407

S15 (TI hydralazin*) OR (TI hydrallazin*) OR (TI hydralizine) OR (TI hydrazinophtalazine) OR (TI hydrazinophthalazine) OR (TI hydrazinophtalizine) OR (TI dralzine) OR (TI hydralacin) OR (TI hydrolazine) OR (TI hypophthalin) OR (TI hypoftalin) OR (TI hydrazinophthalazine) OR (TI idralazina) OR (TI 1-hydrazinophthalazine) OR (TI apressin) OR (TI nepresol) OR (TI apressoline) OR (TI apresoline) OR (TI apresolin) OR (TI alphapress) OR (TI alazine) OR (TI idralazina) OR (TI lopress) OR (TI plethorit) OR (TI praeparat) OR (AB hydralazin*) OR (AB hydrallazin*) OR (AB hydralizine) OR (AB hydrazinophtalazine) OR (AB hydrazinophthalazine) OR (AB hydrazinophtalizine) OR (AB dralzine) OR (AB hydralacin) OR (AB hydrolazine) OR (AB hypophthalin) OR (AB hypoftalin) OR (AB hydrazinophthalazine) OR (AB idralazina) OR (AB 1-hydrazinophthalazine) OR (AB apressin) OR (AB nepresol) OR (AB apressoline) OR (AB apresoline) OR (AB apresolin) OR (AB alphapress) OR (AB alazine) OR (AB idralazina) OR (AB lopress) OR (AB plethorit) OR (AB praeparat) 485

S16 (MH "Adrenergic Beta-Antagonists+") 12094

S17 (TI acebutolol) OR (TI adimolol) OR (TI afurolol) OR (TI alprenolol) OR (TI amosulalol) OR (TI arotinolol) OR (TI atenolol) OR (TI befunolol) OR (TI betaxolol) OR (TI bevantolol) OR (TI bisoprolol) OR (TI bopindolol) OR (TI bornaprolol) OR (TI brefonalol) OR (TI bucindolol) OR (TI bucumolol) OR (TI bufetolol) OR (TI bufuralol) OR (TI bunitrolol) OR (TI bunolol) OR (TI bupranolol) OR (TI butofilolol) OR (TI butoxamine) OR (TI carazolol) OR (TI carteolol) OR (TI carvedilol) OR (TI celiprolol) OR (TI cetamolol) OR (TI chlortalidone cloranolol) OR (TI cyanoiodopindolol) OR (TI cyanopindolol) OR (TI deacetylmetipranolol) OR (TI diacetolol) OR (TI dihydroalprenolol) OR (TI dilevalol) OR (TI epanolol) OR (TI esmolol) OR (TI exaprolol) OR (TI falintolol) OR (TI flestolol) OR (TI flusoxolol) OR (TI hydroxybenzylpinodolol) OR (TI hydroxycarteolol) OR (TI hydroxymetoprolol) OR (TI indenolol) OR (TI iodocyanopindolol) OR (TI iodopindolol) OR (TI iprocrolol) OR (TI isoxaprolol) OR (TI labetalol) OR (TI landiolol) OR (TI levobunolol) OR (TI levomoprolol) OR (TI medroxalol) OR (TI mepindolol) OR (TI methylthiopropranolol) OR (TI metipranolol) OR (TI metoprolol) OR (TI moprolol) OR (TI nadolol) OR (TI oxprenolol) OR (TI penbutolol) OR (TI pindolol) OR (TI nadolol) OR (TI nebivolol) OR (TI nifenalol) OR (TI nipradilol) OR (TI oxprenolol) OR (TI pafenolol) OR (TI pamatolol) OR (TI penbutolol) OR (TI pindolol) OR (TI practolol) OR (TI primidolol) OR (TI prizidilol) OR (TI procinolol) OR (TI pronetalol) OR (TI propranolol) OR (TI proxodolol) OR (TI ridazolol) OR (TI salcardolol) OR (TI soquinolol) OR (TI sotalol) OR (TI spirendolol) OR (TI talinolol) OR (TI tertatolol) OR (TI tienoxolol) OR (TI tilisolol) OR (TI timolol) OR (TI tolamolol) OR (TI toliprolol) OR (TI tribendilol) OR (TI xibenolol) OR (AB acebutolol) OR (AB adimolol) OR (AB afurolol) OR (AB alprenolol) OR (AB amosulalol) OR (AB arotinolol) OR (AB atenolol) OR (AB befunolol) OR (AB betaxolol) OR (AB bevantolol) OR (AB bisoprolol) OR (AB bopindolol) OR (AB bornaprolol) OR (AB brefonalol) OR (AB bucindolol) OR (AB bucumolol) OR (AB bufetolol) OR (AB bufuralol) OR (AB bunitrolol) OR (AB bunolol) OR (AB bupranolol) OR (AB butofilolol) OR (AB butoxamine) OR (AB carazolol) OR (AB carteolol) OR (AB carvedilol) OR (AB celiprolol) OR (AB cetamolol) OR (AB chlortalidone cloranolol) OR (AB cyanoiodopindolol) OR (AB cyanopindolol) OR (AB deacetylmetipranolol) OR (AB diacetolol) OR (AB dihydroalprenolol) OR (AB dilevalol) OR (AB epanolol) OR (AB esmolol) OR (AB exaprolol) OR (AB falintolol) OR (AB flestolol) OR (AB flusoxolol) OR (AB hydroxybenzylpinodolol) OR (AB hydroxycarteolol) OR (AB hydroxymetoprolol) OR (AB indenolol) OR (AB iodocyanopindolol) OR (AB iodopindolol) OR (AB iprocrolol) OR (AB isoxaprolol) OR (AB labetalol) OR (AB landiolol) OR (AB levobunolol) OR (AB levomoprolol) OR (AB medroxalol) OR (AB mepindolol) OR (AB methylthiopropranolol) OR (AB metipranolol) OR (AB metoprolol) OR (AB moprolol) OR (AB nadolol) OR (AB oxprenolol) OR (AB penbutolol) OR (AB pindolol) OR (AB nadolol) OR (AB nebivolol) OR (AB nifenalol) OR (AB nipradilol) OR (AB oxprenolol) OR (AB pafenolol) OR (AB pamatolol) OR (AB penbutolol) OR (AB pindolol) OR (AB practolol) OR (AB primidolol) OR (AB prizidilol) OR (AB procinolol) OR (AB pronetalol) OR (AB propranolol) OR (AB proxodolol) OR (AB ridazolol) OR (AB salcardolol) OR (AB soquinolol) OR (AB sotalol) OR (AB spirendolol) OR (AB talinolol) OR (AB tertatolol) OR (AB tienoxolol) OR (AB tilisolol) OR (AB timolol) OR (AB tolamolol) OR (AB toliprolol) OR (AB tribendilol) OR (AB xibenolol) 6170

S18 (TI beta N1 (adrenergic# or antagonist# or block* or receptor#)) OR (AB beta N1 (adrenergic# or antagonist# or block* or receptor#)) 8716

S19 (MH "Adrenergic Alpha-Antagonists+") 2443

S20 (TI alfuzosin) OR (TI bunazosin) OR (TI doxazosin) OR (TI metazosin) OR (TI neldazosin) OR (TI prazosin) OR (TI silodosin) OR (TI tamsulosin) OR (TI terazosin) OR (TI tiodazosin) OR (TI trimazosin) OR (TI adrenergic N1 (alpha or antagonist#)) OR (TI (adrenergic or alpha or receptor#) N1 block*) OR (AB alfuzosin) OR (AB bunazosin) OR (AB doxazosin) OR (AB metazosin) OR (AB neldazosin) OR (AB prazosin) OR (AB silodosin) OR (AB tamsulosin) OR (AB terazosin) OR (AB tiodazosin) OR (AB trimazosin) OR (AB adrenergic N1 (alpha or antagonist#)) OR (AB (adrenergic or alpha or receptor#) N1 block*) 9240

S21 (MH "Substance Withdrawal, Controlled") 966

S22 (TI deprescri*) OR (TI de-prescri*) OR (TI (withhold* OR withheld OR withdraw* OR discontinu* OR reduced OR reduces OR reducing OR reduction# OR ceased OR ceasing OR cessation# OR stop* OR taper*) N3 (antihypertens* OR anti-hypertens* OR hypotensiv* OR diuretic# OR dosage# OR dose# OR drug* OR medicat* OR prescri* OR treatment# OR therapy OR therapies OR therapeutics)) OR (AB deprescri*) OR (AB de-prescri*) OR (AB (withhold* OR withheld OR withdraw* OR discontinu* OR reduced OR reduces OR reducing OR reduction# OR ceased OR ceasing OR cessation# OR stop* OR taper*) N3 (antihypertens* OR anti-hypertens* OR hypotensiv* OR diuretic# OR dosage# OR dose# OR drug* OR medicat* OR prescri* OR treatment# OR therapy OR therapies OR therapeutics)) 90067

S23 S1 OR S2 1792464

S24 S3 OR S4 141709

S25 S5 OR S6 OR S7 OR S8 OR S9 OR S10 OR S11 OR S12 OR S13 OR S14 OR S15 OR S16 OR S17 OR S18 OR S19 OR S20 76180

S26 S21 OR S22 90815

S27 S23 AND S24 AND S25 AND S26 1056

S28 MH randomized controlled trials 144384

S29 MH double‐blind studies 54095

S30 MH single‐blind studies 16096

S31 MH random assignment 85755

S32 MH pretest‐posttest design 56512

S33 MH cluster sample 5595

S34 TI (randomised OR randomized) 151530

S35 AB (random*) 403554

S36 TI (trial) 194057

S37 MH (sample size) AND AB (assigned OR allocated OR control) 4501

S38 MH (placebos) 14154

S39 PT (randomized controlled trial) 156832

S40 AB (CONTROL W5 GROUP) 149310

S41 MH (CROSSOVER DESIGN) OR MH (COMPARATIVE STUDIES) 496960

S42 AB (CLUSTER W3 RCT) 510

S43 (MH ANIMALS+) NOT (MH HUMAN) 91408

S44 (MH ANIMAL STUDIES) NOT (MH HUMAN) 130697

S45 (TI ANIMAL MODEL*) NOT (MH HUMAN) 3644

S46 S43 OR S44 OR S45 213861

S47 S28 OR S29 OR S30 OR S31 OR S32 OR S33 OR S34 OR S35 OR S36 OR S37 OR S38 OR S39 OR S40 OR S41 OR S42 1051486

S48 S47 NOT S46 1002989

S49 (MH "Cross Sectional Studies") OR (MH "Nonconcurrent Prospective Studies") OR (MH "Prospective Studies") OR (MH "Case Control Studies+") OR (MH "Correlational Studies") 864181

S50 (TI cohort W0 (study OR studies)) OR (AB cohort W0 (study OR studies)) 136314

S51 (TI observational W0 (study OR studies)) OR (AB observational W0 (study OR studies)) 61708

S52 S49 OR S50 OR S51 953700

S53 S48 OR S52 1692705

S54 S27 AND S53 753

S55 (MH "Ocular Hypertension+") OR (MH "Pregnancy-Induced Hypertension+") OR (MH "Pregnancy") OR (MH "Pregnancy Complications, Cardiovascular") 239157

S56 (TI pregnancy) OR (TI "Pregnancy-Induced Hypertension") OR (TI "pregnancy Induced Hypertension") OR (TI "Gestational Hypertension") OR (TI "Pregnancy Transient Hypertension") OR (TI "maternal hypertension") OR (TI "cardiovascular pregnancy complication$") OR (TI "Pregnancy Toxemia$") OR (TI "Edema-Proteinuria-Hypertension Gestosis") OR (TI "Edema Proteinuria Hypertension Gestosis") OR (TI "Hypertension-Edema-Proteinuria Gestosis") OR (TI "Hypertension Edema Proteinuria Gestosis") OR (TI "Toxemia Of Pregnancy") OR (TI "Toxemia Of Pregnancies") OR (TI "EPH Complex") OR (TI "EPH Toxemia$") OR (TI "EPH Gestosis") OR (TI "Proteinuria-Edema-Hypertension Gestosis") OR (TI "Proteinuria Edema Hypertension Gestosis") OR (TI "pre-eclampsia") OR (TI "pre eclampsia") OR (TI "preeclampsia") OR (TI "ocular hypertension$") OR (TI "intraocular hypertension") 77642

S57 S55 OR S56 259995

S58 S54 NOT S57 734

----------------------------------------------------------------------

***************************

Database: Web of Science Core Collection

Entitlements:

- WOS.SCI: 1900 to 2024

- WOS.AHCI: 1975 to 2024

- WOS.ESCI: 2019 to 2024

- WOS.ISTP: 1990 to 2024

- WOS.SSCI: 1900 to 2024

- WOS.ISSHP: 1990 to 2024

Search date: 02/07/2024

Search Strategy:

----------------------------------------------------------------------

1: TS=(advanced years OR ageing OR aging OR elder$ OR elderly OR frail OR geriatric* OR gerontolog* OR later life OR nursing care OR nursing home$ OR old age OR oldest old OR pensioner$ OR post-menopausal OR postmenopausal OR senior OR seniors OR aged OR septuagenarian$ OR octogenarian$ OR nonagenarian$ OR centenarian$ OR supercentenarian$ OR senium) OR TS=(old* NEAR/3 (adult$ OR female$ OR male$ OR men OR people OR person$ OR women OR patient$) ) 6490216

2: TI=((over OR old*) NEAR/2 ("65" OR "66" OR "67" OR "68" OR "69" OR "70" OR "71" OR "72" OR "73" OR "74" OR "75" OR "76" OR "77" OR "78" OR "79" OR "80" OR "81" OR "82" OR "83" OR "84" OR "85" OR "86" OR "87" OR "88" OR "89" OR "90" OR "91" OR "92" OR "93" OR "94" OR "95" OR "96" OR "97" OR "98" OR "99" OR "100") NEAR/2 year$) OR AB=((over OR old*) NEAR/2 ("65" OR "66" OR "67" OR "68" OR "69" OR "70" OR "71" OR "72" OR "73" OR "74" OR "75" OR "76" OR "77" OR "78" OR "79" OR "80" OR "81" OR "82" OR "83" OR "84" OR "85" OR "86" OR "87" OR "88" OR "89" OR "90" OR "91" OR "92" OR "93" OR "94" OR "95" OR "96" OR "97" OR "98" OR "99" OR "100") NEAR/2 year$) 221352

3: SO=(aged OR aging OR ageing OR elder* OR geriatric* OR gerontolog* OR nursing) OR WC=(aged OR aging OR ageing OR elder* OR geriatric* OR gerontolog* OR nursing) OR SU=(aged OR aging OR ageing OR elder* OR geriatric* OR gerontolog* OR nursing) 696159

4: #3 OR #2 OR #1 6832149

5: TS=(hypertens*) OR TS=((elevat* OR high* OR raised) NEAR/2 ("blood pressure" OR "bp") ) 678585

6: TS=(anti$hypertens* OR anti-hypertens* OR hypotensiv* OR thiazide$ OR "sodium chloride symporter inhibitor$" OR "sodium potassium chloride symporter inhibitor$" OR "vasoactive antagonist$" OR vasodilator* OR vasorelaxant$ OR "ganglionic block*" OR "ganglioplegic agent$") 135495

7: TS=((ceiling OR loop) NEAR/1 diuretic$) 4095

8: TS=(amiloride OR benzothiadiazine OR bendroflumethiazide OR bumetanide OR chlorothiazide OR cyclopenthiazide OR furosemide OR hydrochlorothiazide OR hydroflumethiazide OR methyclothiazide OR metolazone OR polythiazide OR trichlormethiazide OR veratide OR chlorthalidone OR chlortalidone OR phthalamudine OR chlorphthalidolone OR oxodoline OR thalitone OR hygroton OR indapamide OR metindamide) 42469

9: TS=((angiotensin NEAR/1 "converting enzyme") NEAR/1 inhibit*) OR TS=((ACE NEAR/1 inhibit*) OR ACEI) OR TS=(arb$ OR ("angiotensin receptor (block* OR antagonist$) ")) 56447

10: TS=(alacepril OR altiopril OR ancovenin OR benazepril OR captopril OR ceranapril OR ceronapril OR cilazapril OR deacetylalacepril OR delapril OR derapril OR enalapril OR epicaptopril OR fasidotril OR fosinopril OR foroxymithine OR gemopatrilat OR idapril OR imidapril OR indolapril OR libenzapril OR lisinopril OR moexipril OR moveltipril OR omapatrilat OR pentopril* OR perindopril* OR pivopril OR quinapril* OR ramipril* OR rentiapril OR saralasin OR s nitrosocaptopril OR spirapril* OR temocapril* OR teprotide OR trandolapril* OR utibapril* OR zabicipril* OR zofenopril* OR Aceon OR Accupril OR Altace OR Capoten OR Lotensin OR Mavik OR Monopril OR Prinivil OR Univas OR Vasotec OR Zestril) 40342

11: TS=(abitesartan OR azilsartan OR candesartan OR elisartan OR embusartan OR eprosartan OR forasartan OR irbesartan OR losartan OR milfasartan OR olmesartan OR saprisartan OR tasosartan OR telmisartan OR valsartan OR zolasartan OR Atacand OR Avapro OR Benicar OR Cozaar OR Diovan OR Micardis OR Teveten) 31899

12: TS=(calcium NEAR/2 (antagonist$ OR block* OR inhibit*) ) 53372

13: TS=(amlodipine OR aranidipine OR barnidipine OR bencyclane OR benidipine OR bepridil OR cilnidipine OR cinnarizine OR clentiazem OR darodipine OR diltiazem OR efonidipine OR elgodipine OR etafenone OR fantofarone OR felodipine OR fendiline OR flunarizine OR gallopamil OR isradipine OR lacidipine OR lercanidipine OR lidoflazine OR lomerizine OR manidipine OR mibefradil OR nicardipine OR nifedipine OR niguldipine OR nilvadipine OR nimodipine OR nisoldipine OR nitrendipine OR perhexiline OR prenylamine OR semotiadil OR terodiline OR tiapamil OR verapamil OR Cardizem CD OR Dilacor XR OR Tiazac OR Cardizem Calan OR Isoptin OR Calan SR OR Isoptin SR Coer OR Covera HS OR Verelan PM) 81459

14: TS=(methyldopa OR alphamethyldopa OR amodopa OR dopamet OR dopegyt OR dopegit OR dopegite OR emdopa OR hyperpax OR hyperpaxa OR methylpropionic acid OR dopergit OR meldopa OR methyldopate OR medopa OR medomet OR sembrina OR aldomet OR aldometil OR aldomin OR hydopa OR methyldihydroxyphenylalanine OR methyl dopa OR mulfasin OR presinol OR presolisin OR sedometil OR sembrina OR taquinil OR dihydroxyphenylalanine OR methylphenylalanine OR methylalanine OR alpha methyl dopa) 10802

15: TS=(reserpine OR serpentina OR rauwolfia OR serpasil) 14900

16: TS=(clonidine OR adesipress OR arkamin OR caprysin OR catapres* OR catasan OR chlofazolin OR chlophazolin OR clinidine OR clofelin* OR clofenil OR clomidine OR clondine OR clonistada OR clonnirit OR clophelin* OR dichlorophenylaminoimidazoline OR dixarit OR duraclon OR gemiton OR haemiton OR hemiton OR imidazoline OR isoglaucon OR klofelin OR klofenil OR m-5041t OR normopresan OR paracefan OR st-155 OR st 155 OR tesno timelets) 24635

17: TS=(hydralazin* OR hydrallazin* OR hydralizine OR hydrazinophtalazine OR hydrazinophthalazine OR hydrazinophtalizine OR dralzine OR hydralacin OR hydrolazine OR hypophthalin OR hypoftalin OR hydrazinophthalazine OR idralazina OR 1-hydrazinophthalazine OR apressin OR nepresol OR apressoline OR apresoline OR apresolin OR alphapress OR alazine OR idralazina OR lopress OR plethorit OR praeparat) 5757

18: TS=("adrenergic beta antagonist$") OR TS=(beta NEAR/2 adrenergic$ NEAR/2 (antagonist$ OR block* OR receptor$) ) 38312

19: TS=(acebutolol OR adimolol OR afurolol OR alprenolol OR amosulalol OR arotinolol OR atenolol OR befunolol OR betaxolol OR bevantolol OR bisoprolol OR bopindolol OR bornaprolol OR brefonalol OR bucindolol OR bucumolol OR bufetolol OR bufuralol OR bunitrolol OR bunolol OR bupranolol OR butofilolol OR butoxamine OR carazolol OR carteolol OR carvedilol OR celiprolol OR cetamolol OR chlortalidone cloranolol OR cyanoiodopindolol OR cyanopindolol OR deacetylmetipranolol OR diacetolol OR dihydroalprenolol OR dilevalol OR epanolol OR esmolol OR exaprolol OR falintolol OR flestolol OR flusoxolol OR hydroxybenzylpinodolol OR hydroxycarteolol OR hydroxymetoprolol OR indenolol OR iodocyanopindolol OR iodopindolol OR iprocrolol OR isoxaprolol OR labetalol OR landiolol OR levobunolol OR levomoprolol OR medroxalol OR mepindolol OR methylthiopropranolol OR metipranolol OR metoprolol OR moprolol OR nadolol OR oxprenolol OR penbutolol OR pindolol OR nadolol OR nebivolol OR nifenalol OR nipradilol OR oxprenolol OR pafenolol OR pamatolol OR penbutolol OR pindolol OR practolol OR primidolol OR prizidilol OR procinolol OR pronetalol OR propranolol OR proxodolol OR ridazolol OR salcardolol OR soquinolol OR sotalol OR spirendolol OR talinolol OR tertatolol OR tienoxolol OR tilisolol OR timolol OR tolamolol OR toliprolol OR tribendilol OR xibenolol) 84876

20: TS=("adrenergic alpha antagonist$") OR TS=(adrenergic NEAR/2 (alpha OR antagonist$) ) OR TS=((adrenergic OR alpha OR receptor$) NEAR/2 block*) 118759

21: TS=(alfuzosin OR bunazosin OR doxazosin OR metazosin OR neldazosin OR prazosin OR silodosin OR tamsulosin OR terazosin OR tiodazosin OR trimazosin) 17352

22: #21 OR #20 OR #19 OR #18 OR #17 OR #16 OR #15 OR #14 OR #13 OR #12 OR #11 OR #10 OR #9 OR #8 OR #7 OR #6 563205

23: TS=(deprescri* OR de-prescri*) 2531

24: TS=((withhold* OR withheld OR withdraw* OR discontinu* OR reduced OR reduces OR reducing OR reduction$ OR ceased OR ceasing OR cessation$ OR stop* OR taper*) NEAR/3 (antihypertens* OR anti-hypertens* OR hypotensiv* OR diuretic$ OR dosage$ OR dose OR doses OR dosing OR drug* OR medicat* OR prescri* OR treatment* OR therapy OR therapies OR therapeutics) ) 439034

25: #24 OR #23 440770

26: #25 AND #22 AND #5 AND #4 3569

27: TI=(pregnancy OR "Gestational Hypertension" OR "maternal hypertension" OR "Edema-Proteinuria-Hypertension Gestosis" OR "Hypertension-Edema-Proteinuria Gestosis" OR "Toxemia Of Pregnancies" OR "EPH Complex" OR "EPH Toxemia$" OR "EPH Gestosis" OR "Proteinuria-Edema-Hypertension Gestosis" OR "Proteinuria Edema Hypertension Gestosis" OR pre-eclampsia OR preeclampsia OR "ocular hypertension$" OR "intraocular hypertension") OR AK=(pregnancy OR "Gestational Hypertension" OR "maternal hypertension" OR "Edema-Proteinuria-Hypertension Gestosis" OR "Hypertension-Edema-Proteinuria Gestosis" OR "Toxemia Of Pregnancies" OR "EPH Complex" OR "EPH Toxemia$" OR "EPH Gestosis" OR "Proteinuria-Edema-Hypertension Gestosis" OR "Proteinuria Edema Hypertension Gestosis" OR pre-eclampsia OR preeclampsia OR "ocular hypertension$" OR "intraocular hypertension") OR KP=(pregnancy OR "Gestational Hypertension" OR "maternal hypertension" OR "Edema-Proteinuria-Hypertension Gestosis" OR "Hypertension-Edema-Proteinuria Gestosis" OR "Toxemia Of Pregnancies" OR "EPH Complex" OR "EPH Toxemia$" OR "EPH Gestosis" OR "Proteinuria-Edema-Hypertension Gestosis" OR "Proteinuria Edema Hypertension Gestosis" OR pre-eclampsia OR preeclampsia OR "ocular hypertension$" OR "intraocular hypertension") 433790

28: TI=(animal$ OR rat OR rats OR mouse OR mice OR pig OR pigs OR murine OR rodent$) OR AK=(animal$ OR rat OR rats OR mouse OR mice OR pig OR pigs OR murine OR rodent$) OR KP=(animal$ OR rat OR rats OR mouse OR mice OR pig OR pigs OR murine OR rodent$) NOT TS=(human$ OR patient$) 3179843

29: #28 OR #27 3586488

30: #26 NOT #29 3042

----------------------------------------------------------------------

***************************

Database: ClinicalTrials.gov

Search date: 02/07/2024

Search results: 461

Search Strategy:

----------------------------------------------------------------------

Other terms: ceased OR ceasing OR cessation OR deprescribe OR deprescribing OR deprescribed OR stop OR stopping OR stopped OR taper OR tapering OR tapered OR withdraw OR withdrawing OR withdrawal OR withdrawn OR discontinuation OR discontinue OR discontinuing

Condition or disease: hypertension

Age: Older adult (65+)

----------------------------------------------------------------------

***************************

Database: ICTRP

Search date: 02/07/2024

Search results: 275

Search Strategy:

----------------------------------------------------------------------

(ceased OR ceasing OR cessation OR deprescribe OR deprescribing OR deprescribed OR stop OR stopping OR stopped OR taper OR tapering OR tapered OR withdraw OR withdrawing OR withdrawal OR withdrawn OR discontinuation OR discontinue OR discontinuing) AND (hypertension OR antihypertensive)

----------------------------------------------------------------------

***************************

Database: Epistemonikos

Search date: 02/07/2024

Search results: 866

Search Strategy:

----------------------------------------------------------------------

(title:(hypertens* OR elevated "blood pressure" OR "elevated bp" OR high "blood pressure" OR "high bp") OR abstract:(hypertens* OR elevated "blood pressure" OR "elevated bp" OR high "blood pressure" OR "high bp")) AND (title:("advanced years" OR ageing OR aging OR elder* OR elderly OR frail OR geriatric* OR gerontology* OR "later life" OR "nursing care" OR "nursing home" OR "nursing homes" OR "old age" OR "oldest old" OR pensioner* OR post‐menopausal OR postmenopausal OR senior*) OR abstract:("advanced years" OR ageing OR aging OR elder* OR elderly OR frail OR geriatric* OR gerontology* OR "later life" OR "nursing care" OR "nursing home" OR "nursing homes" OR "old age" OR "oldest old" OR pensioner* OR post‐menopausal OR postmenopausal OR senior*)) AND (title:(withhold* OR withheld OR withdraw* OR deprescri* OR de-prescri* discontinu* OR reduced OR reduces OR reducing OR reduction* OR ceased OR ceasing OR cessation* OR stop* OR taper*) OR abstract:(withhold* OR withheld OR withdraw* OR deprescri* OR de-prescri* discontinu* OR reduced OR reduces OR reducing OR reduction* OR ceased OR ceasing OR cessation* OR stop* OR taper*)) AND (title:(antihypertens* OR anti-hypertens* OR hypotensiv* OR thiazide* OR "sodium chloride symporter inhibitors" OR "sodium potassium chloride symporter inhibitors" OR "ceiling diuretics" OR "loop diuretics" OR "ceiling diuretic" OR "loop diuretic" OR "angiotensin-converting enzyme inhibitor" OR "angiotensin-converting enzyme inhibitors" OR "ace inhibitor" OR "ace inhibitors" OR acei OR "Angiotensin Receptor Antagonists" OR "Angiotensin Receptor Antagonist" OR "Angiotensin Receptor blocker" OR "Angiotensin Receptor blockers" OR arb OR arbs OR "calcium channel blocker" OR "calcium channel blockers" OR hydralazin* OR hydralacin* OR "adrenergic beta-antagonist" OR "adrenergic beta-antagonists" OR "beta blocker" OR "beta blockers" OR "adrenergic alpha antagonist" OR "adrenergic alpha antagonists" OR vasodilator* OR vasorelaxant*) OR abstract:(antihypertens* OR anti-hypertens* OR hypotensiv* OR thiazide* OR "sodium chloride symporter inhibitors" OR "sodium potassium chloride symporter inhibitors" OR "ceiling diuretics" OR "loop diuretics" OR "ceiling diuretic" OR "loop diuretic" OR "angiotensin-converting enzyme inhibitor" OR "angiotensin-converting enzyme inhibitors" OR "ace inhibitor" OR "ace inhibitors" OR acei OR "Angiotensin Receptor Antagonists" OR "Angiotensin Receptor Antagonist" OR "Angiotensin Receptor blocker" OR "Angiotensin Receptor blockers" OR arb OR arbs OR "calcium channel blocker" OR "calcium channel blockers" OR hydralazin* OR hydralacin* OR "adrenergic beta-antagonist" OR "adrenergic beta-antagonists" OR "beta blocker" OR "beta blockers" OR "adrenergic alpha antagonist" OR "adrenergic alpha antagonists" OR vasodilator* OR vasorelaxant*))

----------------------------------------------------------------------

1. **Outcome summary table**

| **Outcome** | **N of studies** | **N of participants** | **Comparison** | **Pooled Effect Estimate** | **Heterogeneity (I^2^)** |
| --- | --- | --- | --- | --- | --- |
| All-cause mortality | 6 | 1,590 | Deprescribing antihypertensive vs Continuation | OR 1.11 (95% CI 0.82-1.50) | 0.40 |
| Myocardial infarction | 3 | 1,159 | Deprescribing antihypertensive vs Continuation | 1.32 (95% CI 0.30-5.92) | 0.40 |
| Heart failure | 3 | 385 | Deprescribing antihypertensive vs Continuation | 3.16 (95% CI 1.53-6.55) | 0.40 |
| Stroke | 4 | 1,410 | Deprescribing antihypertensive vs Continuation | OR of 3.08 (95% CI 0.73-13.00) | 0.40 |

CI= Confidence Interval; MACE= Major Adverse Cardiovascular Events; N= Number; OR= Odds Ratio

1. **Glossary of subjective endpoints**

**MACE, Major Cardiovascular Events:** major cardiovascular events were defined as hospital admission for non-fatal stroke, myocardial infarction, heart failure, or cardiovascular mortality using ICD-10 codes reported in the electronic health care records (1)

**Orthostatic Hypotension:** Orthostatic hypotension was defined as a drop of 10 mmHg or more in diastolic blood pressure or 20 mmHg or more in systolic blood pressure (2)

1. **Subgroup Analyses considering duration of follow up**

Forest plot all-cause mortality


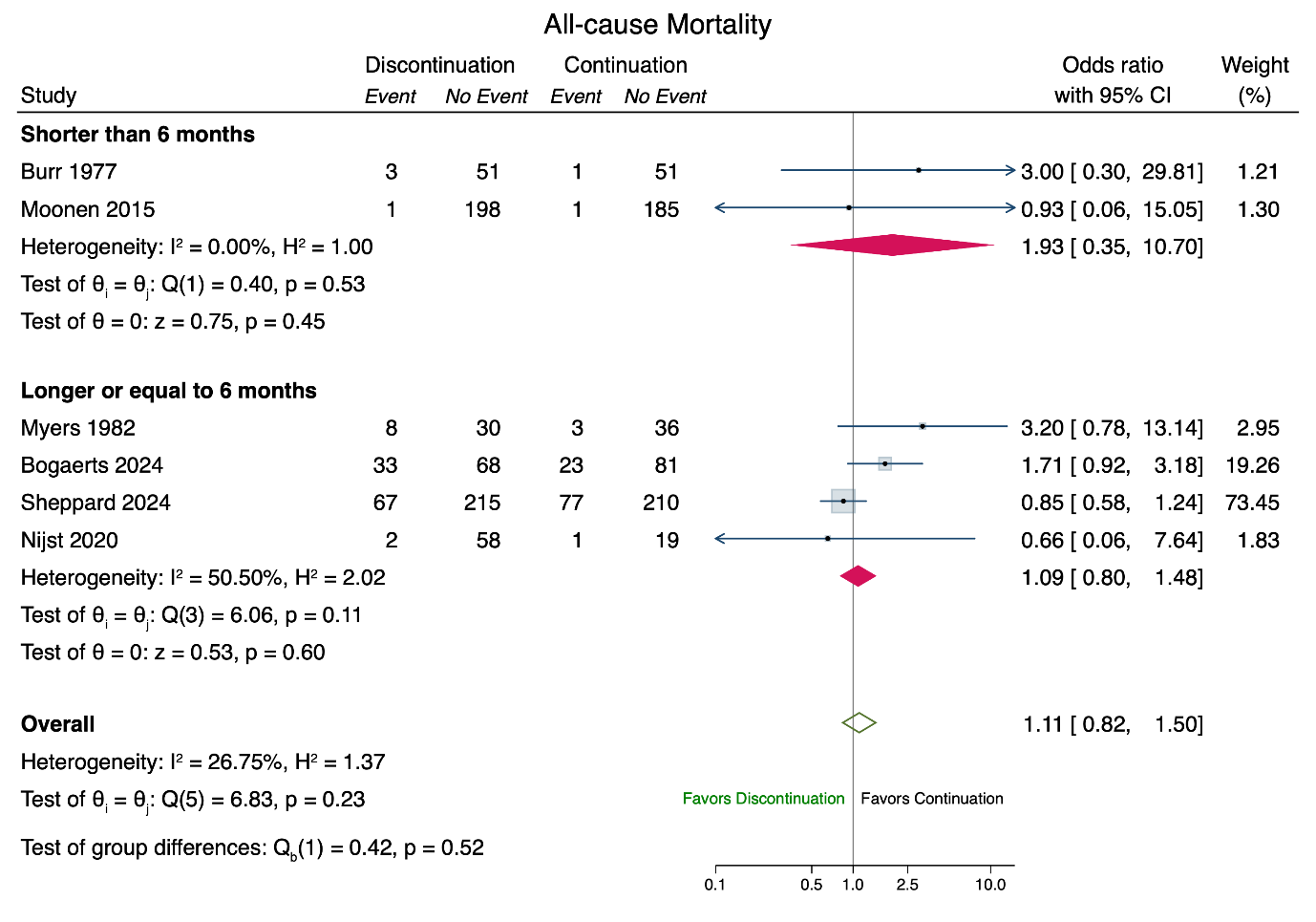


Forest plot myocardial infarction


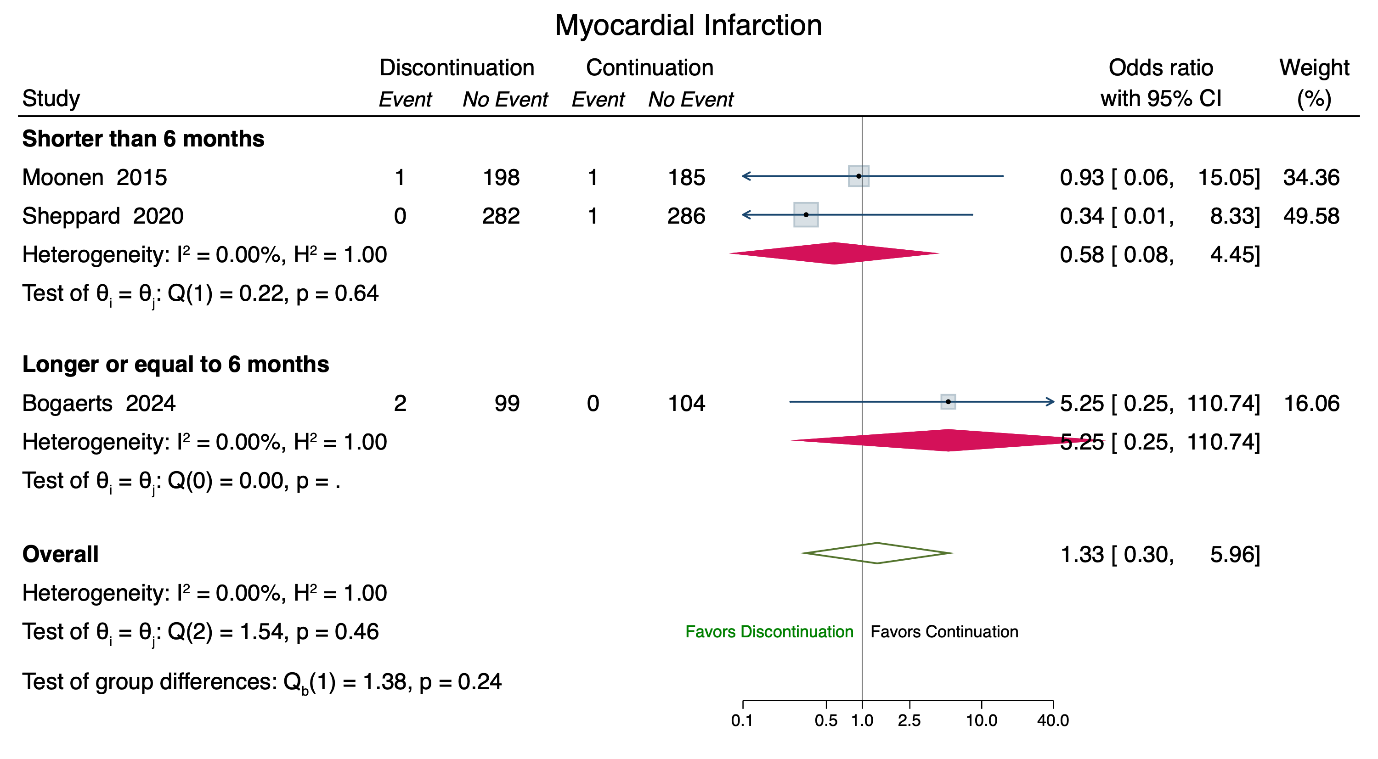


Forest plot heart failure

**
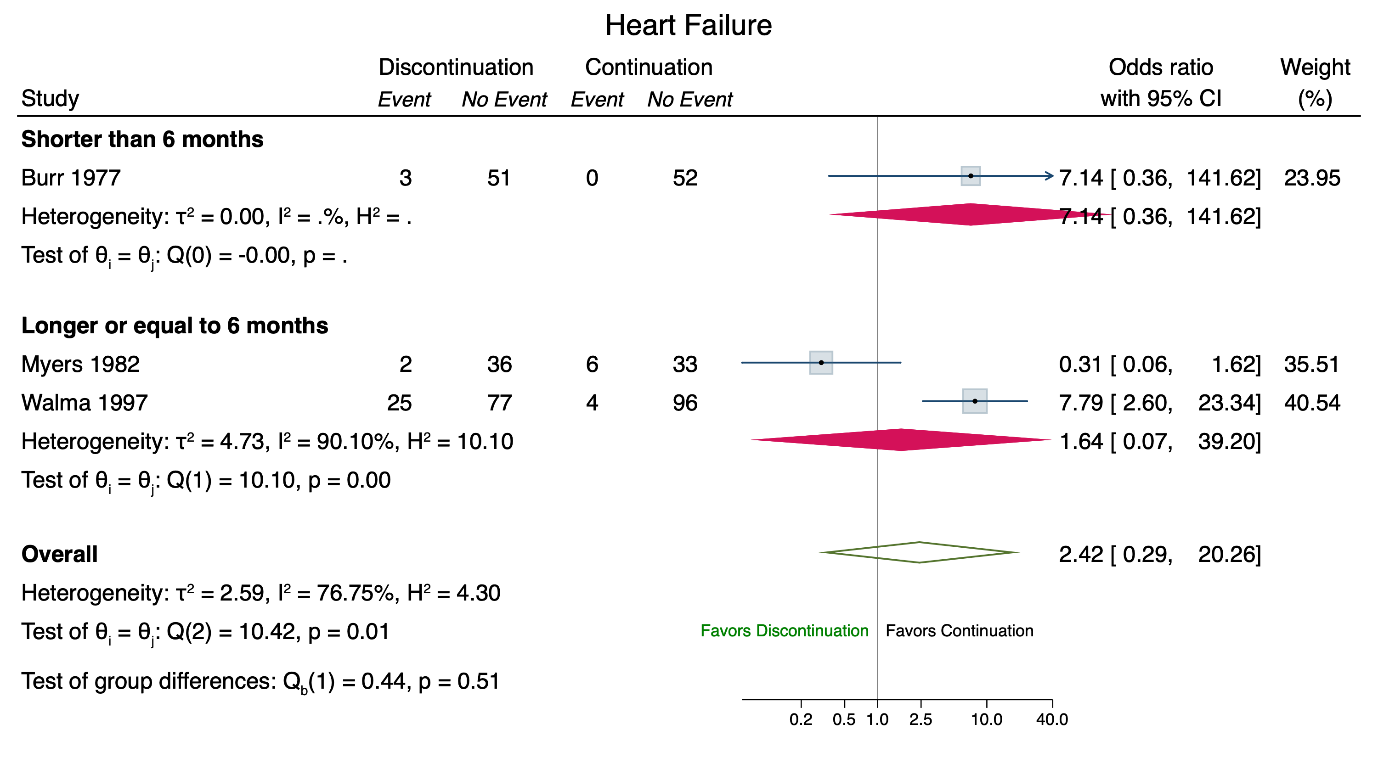
**

Forest plot stroke


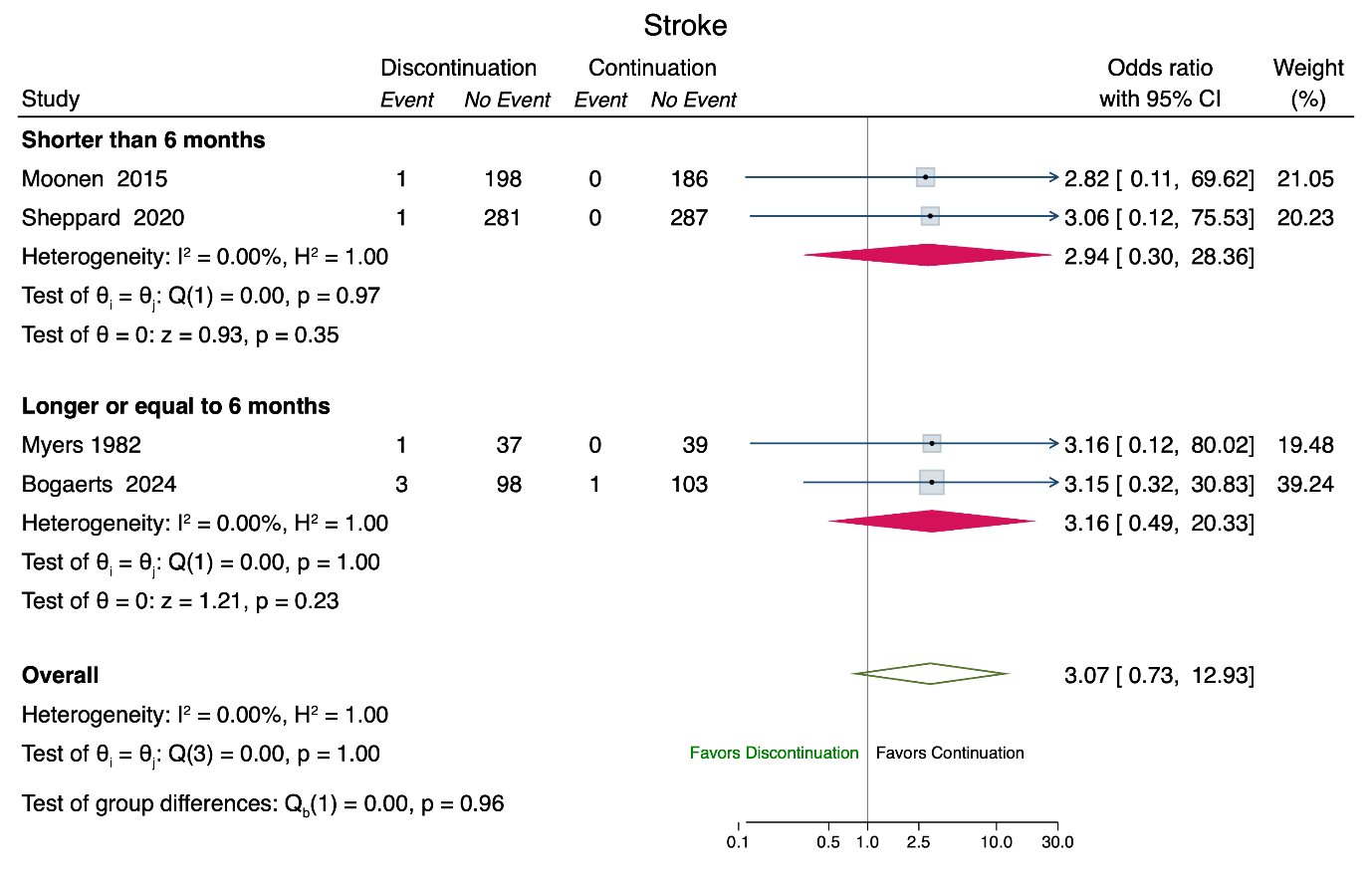


1. **Sensitivity Analisys with ramdom-effect meta-analysis**

Forest plot all-cause mortality


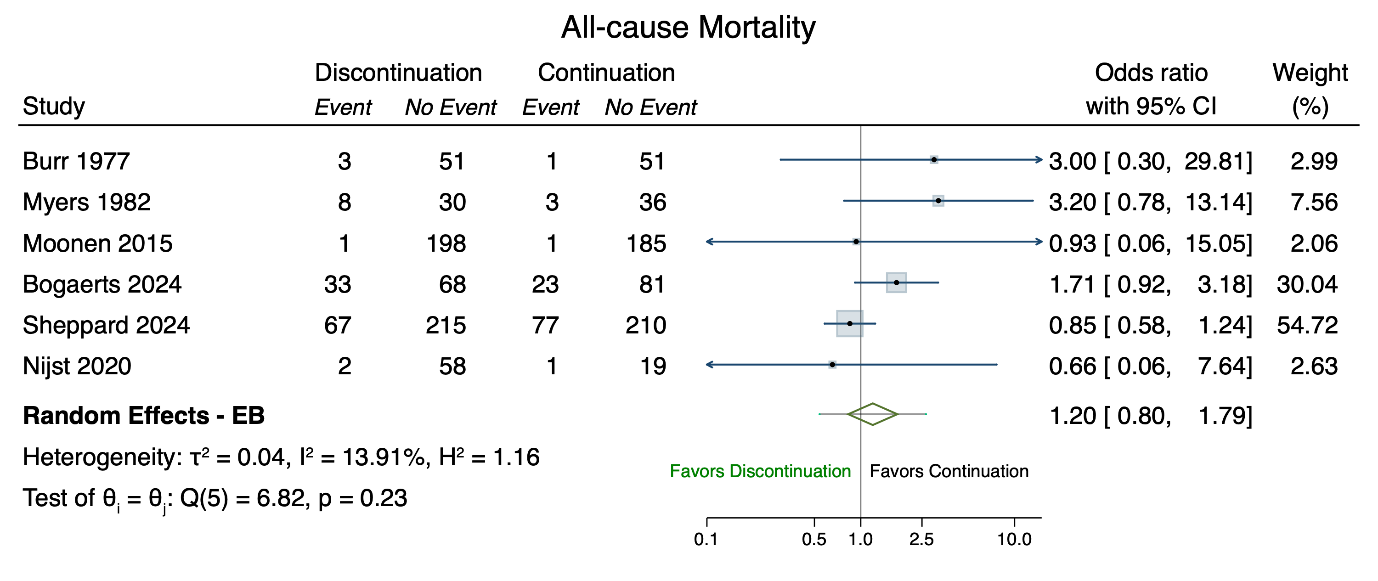


Forest plot my ocardial infarction
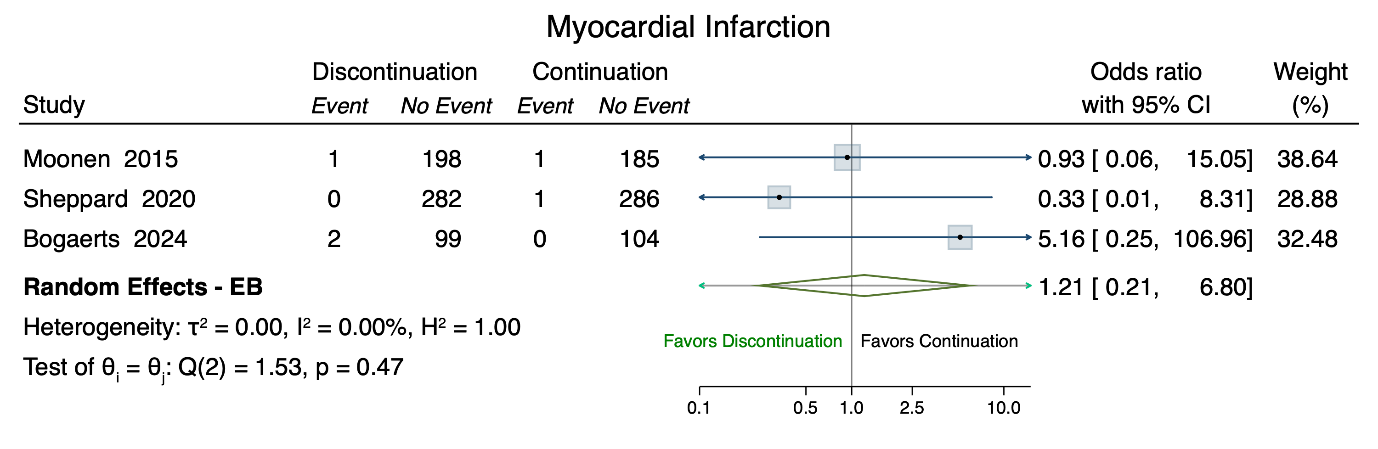


Forest plot heart failure


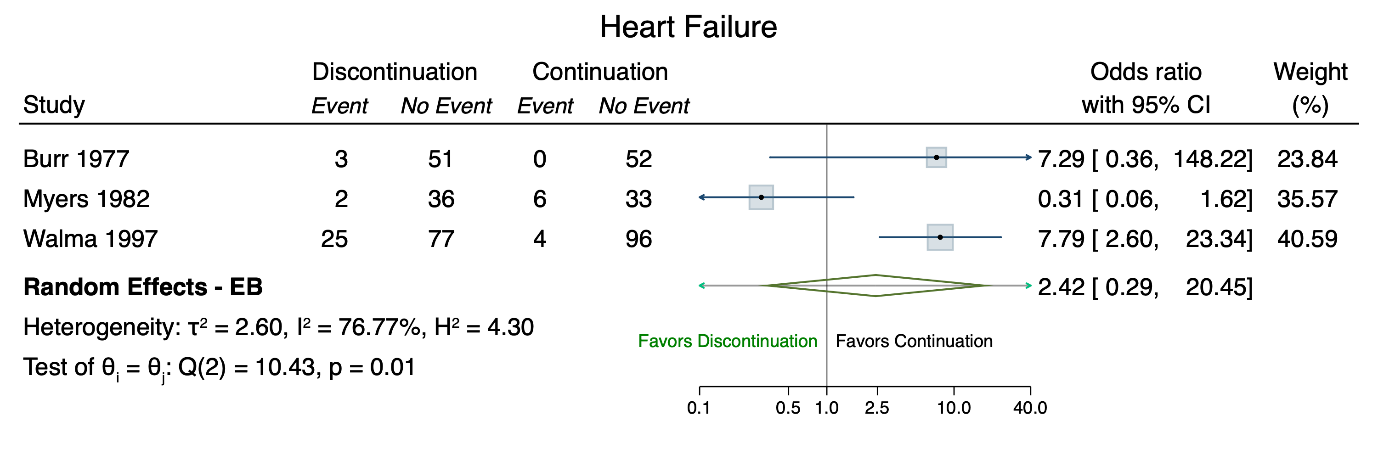


Forest plot stroke


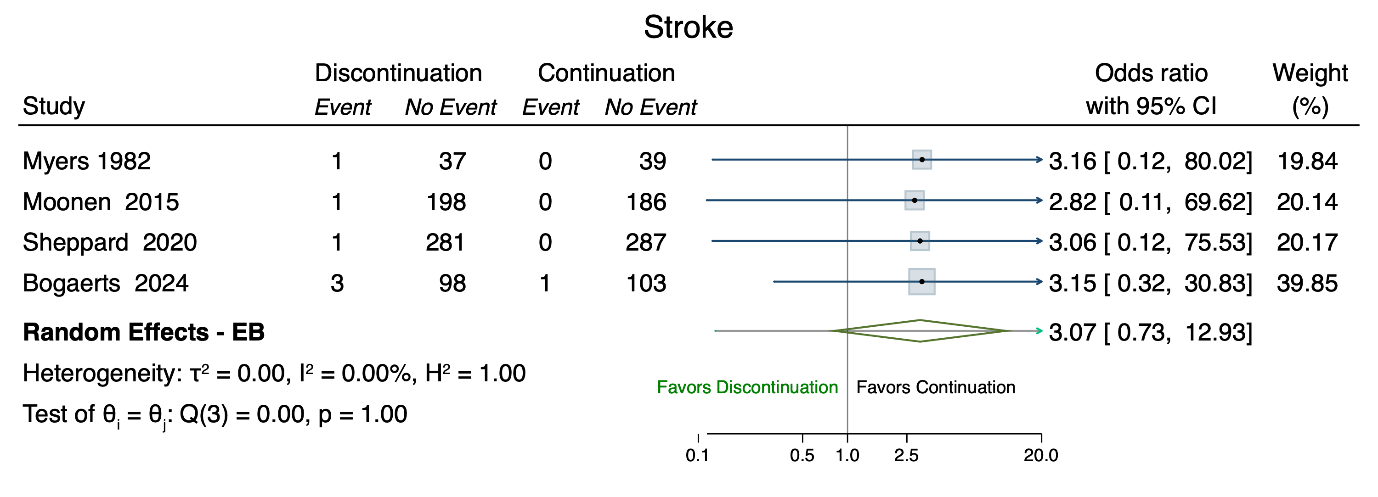


1. **Risk of publication bias or small study effect**

All-cause mortality


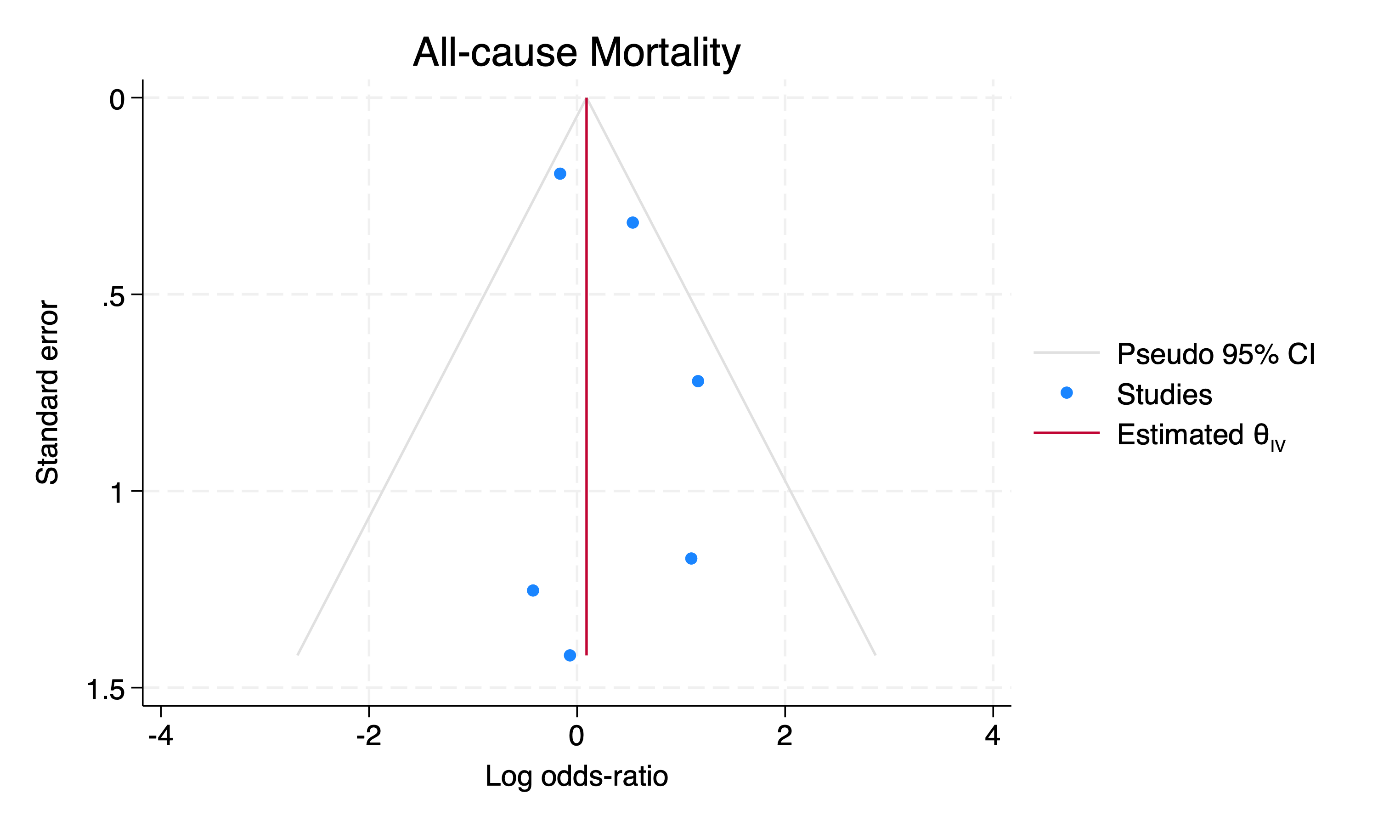


Myocardial infarction


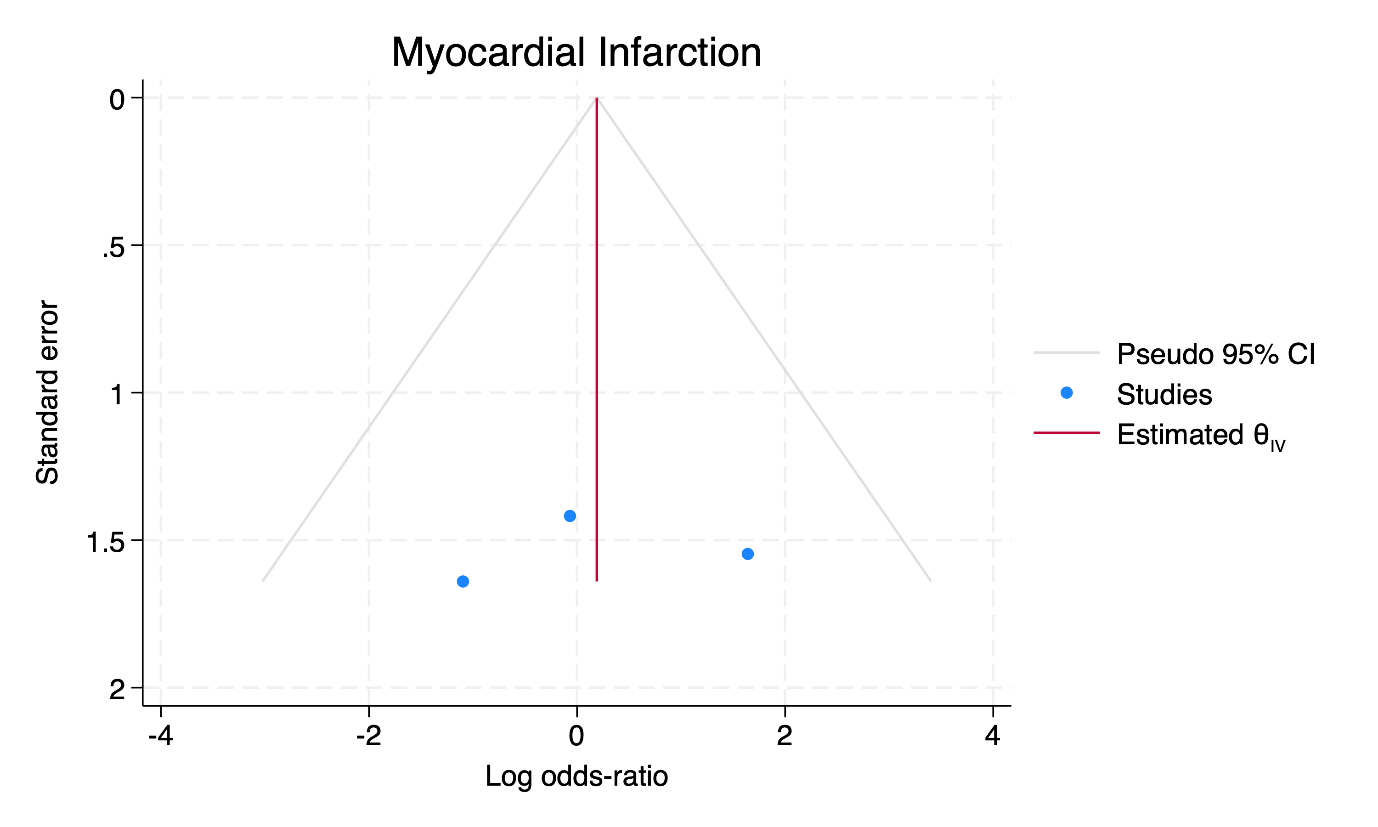


Heart failure


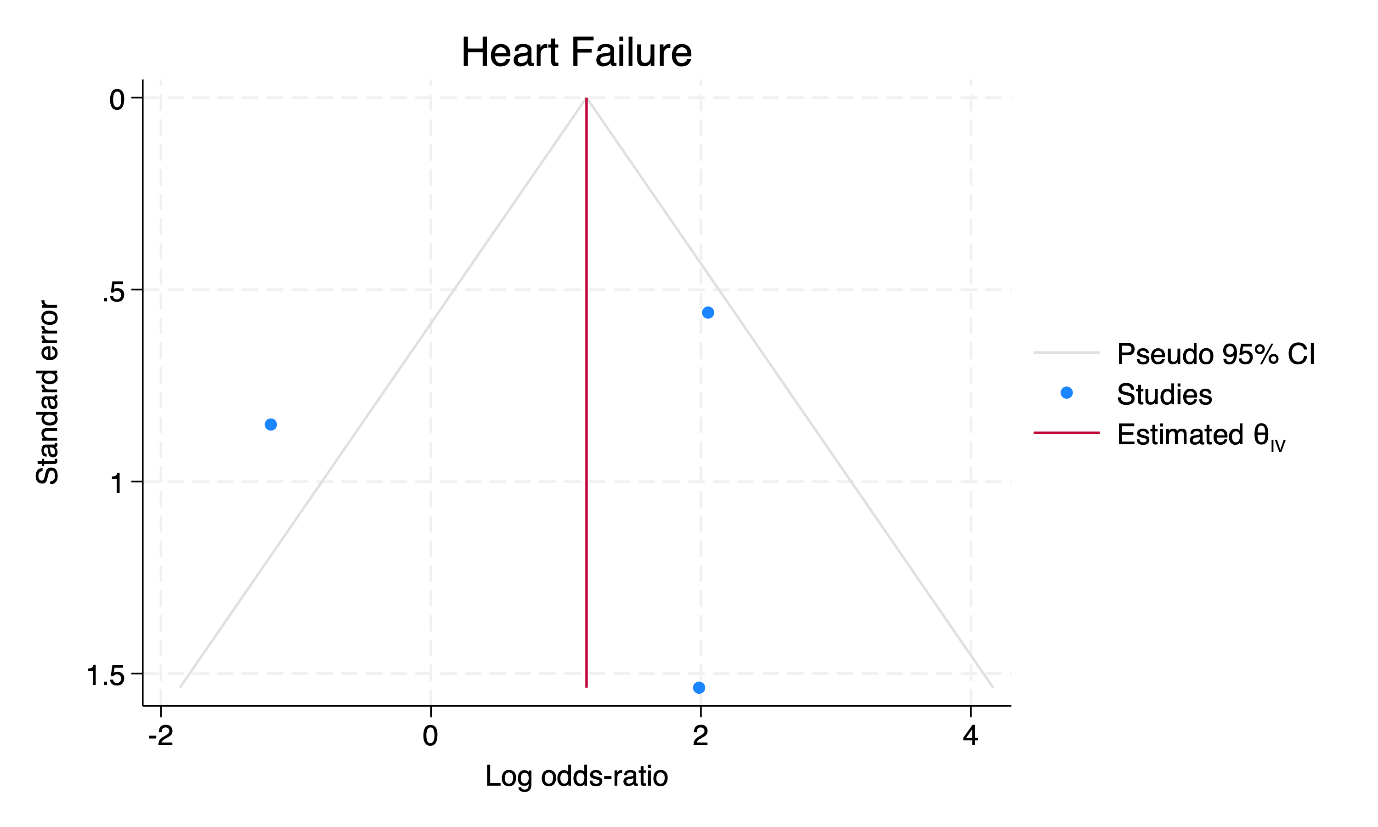


Stroke

**
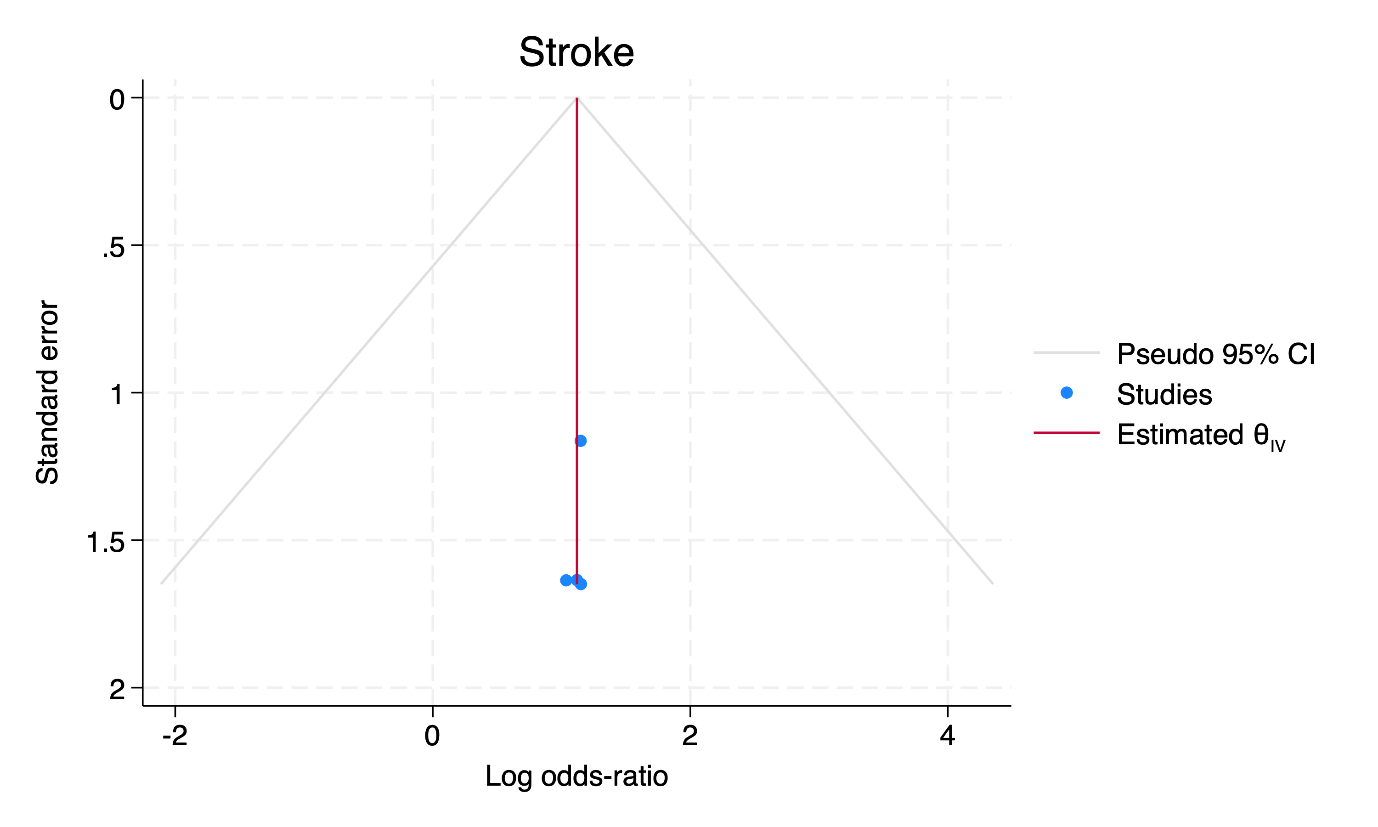
**

1. **Quality assessment of observational studies according to the Newcastle Ottawa Scale for cohort studies**

| **Title/Author** | **Selection** | **Comparabiliy** | **Outcome** | **Overall Quality** |
| --- | --- | --- | --- | --- |
| Antihypertensive Drug Deintensification and Recurrent Falls in Long-Term Care  Wei Song et al. (3) | **Representativeness exposed cohort** | **Comparability of cohorts based on the design or analysis controlled for confounders** | **Assessment of outcome** | **Good** |
|  | b) Somewhat representative ***(one star)*** | b) Study controls for other factors (list) ***(one star)*** | b) Record linkage ***(one star)*** |  |
|  | **Selection of non-exposed cohort** |  | **Was follow-up long enough for outcomes to occur** |  |
|  | b) Drawn from a different source |  | a) Yes ***(one star)*** |  |
|  | **Ascertainment of exposure** |  | **Adequacy of follow-up of cohorts** |  |
|  | a) Secure record (e.g., surgical record) ***(one star)*** |  | b) Subjects lost to follow up unlikely to introduce bias- number lost less than or equal to 20% or description of those lost suggested no different from those followed. ***(one star)*** |  |
|  | **Demonstration that outcome of interest was not present at start of study** |  |  |  |
|  | a) Yes ***(one star)*** |  |  |  |

| **Title/Author** | **Selection** | **Comparability** | **Outcome** | **Overall Quality** |
| --- | --- | --- | --- | --- |
| Stopping Renin-Angiotensin System Inhibitors  in patients with advanced CKD and risk of adverse outcomes: a nationwide study  Eduard L Fu et al. (4) | **Representativeness exposed cohort** | **Comparability of cohorts based on the design or analysis controlled for confounders** | **Assessment of outcome** | **Good** |
|  | c) Selected group | b) Study controls for other factors (list) ***(one star)*** | b) Record linkage ***(one star)*** |  |
|  | **Selection of non-exposed cohort** |  | **Was follow-up long enough for outcomes to occur** |  |
|  | a) Drawn from the same community as the exposed cohort ***(one star)*** |  | a) Yes ***(one star)*** |  |
|  | **Ascertainment of exposure** |  | **Adequacy of follow-up of cohorts** |  |
|  | a) Secure record (e.g., surgical record) ***(one star)*** |  | d) No statement |  |
|  | **Demonstration that outcome of interest was not present at start of study** |  |  |  |
|  | a) Yes ***(one star)*** |  |  |  |

| **Title/Author** | **Selection** | **Comparability** | **Outcome** | **Overall Quality** |
| --- | --- | --- | --- | --- |
| Association between  Renin-Angiotensin System Blockade Discontinuation and all-cause mortality among persons with low estimated glomerular filtration rate  Yao Qiao et al. (5) | **Representativeness exposed cohort** | **Comparability of cohorts based on the design or analysis controlled for confounders** | **Assessment of outcome** | **Good** |
|  | a) Truly representative ***(one star)*** | b) Study controls for other factors (list) ***(one star)*** | b) Record linkage ***(one star)*** |  |
|  | **Selection of non-exposed cohort** |  | **Was follow-up long enough for outcomes to occur** |  |
|  | a) Drawn from the same community as the exposed cohort ***(one star)*** |  | a) Yes ***(one star)*** |  |
|  | **Ascertainment of exposure** |  | **Adequacy of follow-up of cohorts** |  |
|  | a) Secure record (e.g., surgical record) ***(one star)*** |  | d) No statement |  |
|  | **Demonstration that outcome of interest was not present at start of study** |  |  |  |
|  | a) Yes ***(one star)*** |  |  |  |

| **Title/Author** | **Selection** | **Comparability** | **Outcome** | **Overall Quality** |
| --- | --- | --- | --- | --- |
| Discontinuation of beta-blockers  and the risk of myocardial infarction in the elderly  Teichert et al. (6) | **Representativeness exposed cohort** | **Comparability of cohorts based on the design or analysis controlled for confounders** | **Assessment of outcome** | **Good** |
|  | a) Truly representative ***(one star)*** | b) Study controls for other factors (list) ***(one star)*** | a) Independent blind assessment ***(one star)*** |  |
|  | **Selection of non-exposed cohort** |  | **Was follow-up long enough for outcomes to occur** |  |
|  | a) Drawn from the same community as the exposed cohort ***(one star)*** |  | a) Yes ***(one star)*** |  |
|  | **Ascertainment of exposure** |  | **Adequacy of follow-up of cohorts** |  |
|  | a) Secure record (e.g., surgical record) ***(one star)*** |  | d) No statement |  |
|  | **Demonstration that outcome of interest was not present at start of study** |  |  |  |
|  | a) Yes ***(one star)*** |  |  |  |

| **Title/Author** | **Selection** | **Comparability** | **Outcome** | **Overall Quality** |
| --- | --- | --- | --- | --- |
| Discontinuation  of Antihypertensive Medication, Cognitive Complaints, and Incident Dementia  van Dalen Willem et al. (7) | **Representativeness exposed cohort** | **Comparability of cohorts based on the design or analysis controlled for confounders** | **Assessment of outcome** | **Good** |
|  | a) Truly representative ***(one star)*** | b) Study controls for other factors (list) ***(one star)*** | a) Independent blind assessment ***(one star)*** |  |
|  | **Selection of non-exposed cohort** |  | **Was follow-up long enough for outcomes to occur** |  |
|  | a) Drawn from the same community as the exposed cohort ***(one star)*** |  | a) Yes ***(one star)*** |  |
|  | **Ascertainment of exposure** |  | **Adequacy of follow-up of cohorts** |  |
|  | b) Structured interview ***(one star)*** |  | c) Follow up rate less than 80% and no description of those lost |  |
|  | **Demonstration that outcome of interest was not present at start of study** |  |  |  |
|  | a) Yes ***(one star)*** |  |  |  |

| **Title/Author** | **Selection** | **Comparability** | **Outcome** | **Overall Quality** |
| --- | --- | --- | --- | --- |
| Clinical outcomes of modifying hypertension treatment intensity in older adults treated to low blood pressure  Aubert et al. (8) | **Representativeness exposed cohort** | **Comparability of cohorts based on the design or analysis controlled for confounders** | **Assessment of outcome** | **Good** |
|  | b) somewhat representative ***(one star)*** | b) Study controls for other factors age, chronic conditions, baseline SBP, baseline antihypertensive medication dose (one star) | b) record linkage (one star) |  |
|  | **Selection of non-exposed cohort** |  | **Was follow-up long enough for outcomes to occur** |  |
|  | a) Drawn from the same community as the exposed cohort ***(one star)*** |  | a) Yes ***(one star)*** |  |
|  | **Ascertainment of exposure** |  | **Adequacy of follow-up of cohorts** |  |
|  | a)Secure record) ***one star)*** |  | c) Follow up rate less than 80% and no description of those lost |  |
|  | **Demonstration that outcome of interest was not present at start of study** |  |  |  |
|  | a) Yes ***(one star)*** |  |  |  |

| **Title/Author** | **Selection** | **Comparability** | **Outcome** | **Overall Quality** |
| --- | --- | --- | --- | --- |
| Effects of discontinuation of antihypertensive drugs on frailty syndrome in outpatients  Hasegawa et al. (9) | **Representativeness exposed cohort** | **Comparability of cohorts based on the design or analysis controlled for confounders** | **Assessment of outcome** | **Poor** |
|  | b) Somewhat representative ***(one star)*** | d) no description | b) record linkage |  |
|  | **Selection of non-exposed cohort** |  | **Was follow-up long enough for outcomes to occur** |  |
|  | a) Drawn from the same community as the exposed cohort ***(one star)*** |  | a) Yes ***(one star)*** |  |
|  | **Ascertainment of exposure** |  | **Adequacy of follow-up of cohorts** |  |
|  | a)Secure record) ***one star)*** |  | d) No statement |  |
|  | **Demonstration that outcome of interest was not present at start of study** |  |  |  |
|  | a) Yes ***(one star)*** |  |  |  |

1. **Adjustment for confounding factors in the included observational studies**

| Antihypertensive Drug Deintensification and Recurrent Falls in Long-Term Care  Wei Song et al. (3) | Age, disease burden, cognitive and physical function, comorbidties, use of antibiotics |
| --- | --- |
| Stopping Renin-Angiotensin System Inhibitors  in patients with advanced CKD and risk of adverse outcomes: a nationwide study  Eduard L Fu et al. (4) | Age, sex, diabetes mellitus, heart failure |
| Association between  Renin-Angiotensin System Blockade Discontinuation and all-cause mortality among persons with low estimated glomerular filtration rate  Yao Qiao et al. (5) | Age, sex, ethnicity, time of GFR decrease |
| Discontinuation of beta-blockers  and the risk of myocardial infarction in the elderly  Teichert et al. (6) | Age, gender, comorbid heart failure, current use of antihypertensives other that beta-blockers, prevalence of asthma and chronic obstructive disease |
| Discontinuation  of Antihypertensive Medication, Cognitive Complaints, and Incident Dementia  van Dalen Willem et al. (7) | Age, sex, baseline systolic blood pressure, total number of antihypertensives, total numebre of medications, history of stroke, history cardiovascular disease, subjective memory complaints |
| Clinical outcomes of modifying hypertension treatment intensity in older adults treated to low blood pressure  Aubert et al. (8) | Extensive adjustments, under other for age, chronic conditions, baseline systolic blood pressure, baseline antihypertensive medications |
| Effects of discontinuation of antihypertensive drugs on frailty syndrome in outpatients  Hasegawa et al. (9) | No information on adjustment procedures |

**Bibliography**

1. Sheppard JP, Temple E, Wang A, Smith A, Pollock S, Ford GA, et al. Effect of antihypertensive deprescribing on hospitalisation and mortality: long-term follow-up of the OPTiMISE randomised controlled trial. Lancet Healthy Longev. 2024 Aug;5(8):e563–73.

2. Moonen JEF, Foster-Dingley JC, de Ruijter W, van der Grond J, de Craen AJM, van der Mast RC. Effect of discontinuation of antihypertensive medication on orthostatic hypotension in older persons with mild cognitive impairment: the DANTE Study Leiden. Age Ageing. 2016 Mar;45(2):249–55.

3. Song W, Intrator O, Lee S, Boockvar K. Antihypertensive Drug Deintensification and Recurrent Falls in Long-Term Care. Health Serv Res. 2018 Dec;53(6):4066–86.

4. Fu EL, Evans M, Clase CM, Tomlinson LA, van Diepen M, Dekker FW, et al. Stopping Renin-Angiotensin System Inhibitors in Patients with Advanced CKD and Risk of Adverse Outcomes: A Nationwide Study. J Am Soc Nephrol JASN. 2021 Feb;32(2):424–35.

5. Qiao Y, Shin JI, Chen TK, Inker LA, Coresh J, Alexander GC, et al. Association Between Renin-Angiotensin System Blockade Discontinuation and All-Cause Mortality Among Persons With Low Estimated Glomerular Filtration Rate. JAMA Intern Med. 2020 May 1;180(5):718–26.

6. Teichert M, de Smet PAGM, Hofman A, Witteman JCM, Stricker BHC. Discontinuation of beta-blockers and the risk of myocardial infarction in the elderly. Drug Saf. 2007;30(6):541–9.

7. van Dalen JW, Moll van Charante EP, van Gool WA, Richard E. Discontinuation of Antihypertensive Medication, Cognitive Complaints, and Incident Dementia. J Am Med Dir Assoc. 2019 Sep;20(9):1091-1097.e3.

8. Aubert CE, Ha J, Kim HM, Rodondi N, Kerr EA, Hofer TP, et al. Clinical outcomes of modifying hypertension treatment intensity in older adults treated to low blood pressure. J Am Geriatr Soc. 2021 Jun 7;jgs.17295.

9. Hasegawa S, Mizokami F, Mase H, Hayakawa Y, Shimizu A, Matsui Y. Effects of discontinuation of antihypertensive drugs on frailty syndrome in outpatients: a 1-year prospectively designed retrospective chart-review pilot study. J Int Med Res. 2022 Oct;50(10):03000605221130716.
